# Red Foxes (*Vulpes vulpes*) and European Badgers (*Meles meles*) as Overlooked Wildlife Hosts of Canine Parvovirus in Slovakia: First Evidence by Molecular Characterization and Virus Isolation

**DOI:** 10.3390/microorganisms13102325

**Published:** 2025-10-08

**Authors:** Patrícia Petroušková, Andrea Pelegrinová, Jozef Lazár, Jakub Lipinský, Monika Drážovská, Marián Prokeš, Ľuboš Korytár, Boris Vojtek, Maroš Kostičák, Ladislav Molnár, Jana Mojžišová Vaščinec, Anna Ondrejková

**Affiliations:** 1 Department of Epizootiology, Parasitology and Protection of One Health, University of Veterinary Medicine and Pharmacy in Košice, Komenského 73, 041 81 Košice, Slovakia; andrea.pelegrinova@student.uvlf.sk (A.P.); jakub.lipinsky@student.uvlf.sk (J.L.); monika.drazovska@uvlf.sk (M.D.); marian.prokes@uvlf.sk (M.P.); lubos.korytar@uvlf.sk (Ľ.K.); boris.vojtek@uvlf.sk (B.V.); maros.kosticak@student.uvlf.sk (M.K.); jana.mojzisova@uvlf.sk (J.M.V.); anna.ondrejkova@uvlf.sk (A.O.); 2 Department of Breeding and Diseases of Game, Fish and Bees, Ecology and Cynology, University of Veterinary Medicine and Pharmacy in Košice, Komenského 73, 041 81 Košice, Slovakia; jozef.lazar@uvlf.sk; 3 Clinic of Birds, Exotic and Free Living Animals, University of Veterinary Medicine and Pharmacy in Košice, Komenského 73, 041 81 Košice, Slovakia; ladislav.molnar@uvlf.sk

**Keywords:** canine parvovirus, Slovakia, wildlife, red fox, European badger, spillover, molecular characterization, phylogeny, virus isolation

## Abstract

Wild carnivores are increasingly recognized as hosts or reservoirs of canine parvovirus (CPV), a major pathogen of dogs. To investigate CPV circulation in Central Europe, we examined 221 red foxes (*Vulpes vulpes*) and 53 European badgers (*Meles meles*) from seven Slovakian regions. Small intestines (*n* = 86), rectal swabs (*n* = 123), and feces (*n* = 65) were tested by real-time PCR, and positives were sequenced for the *VP2* gene. Virus isolation was performed on MDCK cells. CPV was detected in 10.9% (30/274) of samples, with a prevalence of 10.9% (24/221) in foxes and 11.3% (6/53) in badgers. Phylogenetic analysis revealed co-circulation of CPV-2a, CPV-2b, and CPV-2c, with CPV-2b being prevalent (20/30, 66.7%) and CPV-2c detected in a single sample (1/30, 3.3%). CPV-2a/2b isolates clustered with European strains, while CPV-2c grouped within the “Asian” lineage. Several sequences carried “Asian-like” signatures (5G, 267Y, 324I, 370R), suggesting transboundary introduction and spillover into wildlife. Two infrequent substitutions were identified: S552I in CPV-2b and I447M in CPV-2a. Viable virus was isolated from all positives, with characteristic CPV-cytopathic effects. This study provides the first molecular and virological evidence of CPV in Central European wildlife. Our findings expand our understanding of CPV diversity in Europe, and underscore wildlife as an integral component of European ecology.

## 1. Introduction

Canine parvovirus (CPV) is one of the most important viral pathogens of domestic dogs, causing acute and often fatal enteritis, particularly in puppies and immunologically naive animals [1]. Since its first emergence in the mid-1970s [2], the original CPV type 2 (CPV-2) has undergone notable antigenic drift, consecutively giving rise to new variants that have spread rapidly worldwide, becoming a major veterinary and ecological concern due to their high morbidity and long environmental persistence [3,4].

Taxonomically, CPV belongs to the species *Carnivore protoparvovirus 1*, genus *Protoparvovirus*, and family Parvoviridae. Parvoviruses are characterized by a small, non-enveloped, single-stranded DNA genome, encoding two open reading frames (ORFs), among which ORF1 encodes the non-structural proteins NS1 and NS2 and ORF2 encodes the capsid proteins VP1 and VP2 [5,6]. Despite its relatively simple genetic structure, CPV exhibits remarkable evolutionary potential. The genome of parvoviruses is known to mutate at a higher rate than most other DNA viruses (about 10^−4^ mutations per nucleotide per year). For instance, human parvovirus B19 has been estimated to evolve at 1.03 (0.6–1.27) × 10^−4^ [7], and porcine parvoviruses show similar values ranging from 3.86 × 10^−4^ to 8.23 × 10^−4^ substitutions per site per year [8]. Likewise, feline panleukopenia virus (FPV) was reported to evolve at approximately 1.13 × 10^−4^ substitutions per site per year in the *VP2* gene [9]. In line with these estimates, CPV also exhibits a high substitution rate, approximating 1.7 × 10^−4^ per site per year [4,10,11], comparable to many RNA viruses. This genetic variability has led to the emergence of several antigenic and genetic variants (CPV-2a, -2b, -2c, new CPV-2a, new CPV-2b), which have gradually replaced the original CPV-2 type [11,12,13,14,15]. Mutations within the gene encoding the major capsid protein VP2 influence virus–host cell interactions, virulence, receptor binding, vaccine escape potential, adaptation to novel ecological niches, and host range [3,16,17,18].

Although dogs (*Canis lupus familiaris*) are considered a primary host, CPV has been documented in a broad spectrum of wild animals, especially members of the order Carnivora. These include both wild canids, such as red foxes, wolves, or coyotes [19,20,21,22,23,24,25,26,27], and non-canid species such as felids (cats, pumas, and civets) [22,28,29,30,31], mustelids (badgers and martens) [19,21,22], and procyonids (raccoons) [32]. Molecular studies based on the *VP2* sequencing have shown that parvoviruses from wild and domestic carnivores are genetically identical or closely related, providing strong evidence for cross-species transmission [33]. Transmission occurs primarily via the fecal–oral route, with viral particles remaining infectious in the environment for at least five months under favorable conditions (e.g., shaded, moist environments) [34]. Such stability to external environmental conditions allows for indirect transmission across species and habitats, with wild animals becoming potential sentinel hosts [35]. In wildlife hosts, the pathogenicity of parvovirus infection ranges from asymptomatic infection to severe diarrhea, which may have an impact on the health of vulnerable populations [19,21,22].

The dynamic of CPV transmission is influenced by ecological interactions at the wildlife–domestic interface. Domestic dogs often overlap with wild carnivores as competitors, predators, or prey [36]. On the other hand, wildlife species can act as reservoirs of CPV infection and thereby facilitate the viral persistence in the environment and disseminate the virus to domestic canine populations [37]. Habitat loss or its fragmentation increases the chances of wildlife–domestic contact, as many carnivores adapt to human-modified urban, peri-urban, and rural environments, where they encounter anthropogenic food sources and virus-contaminated fomites and establish dens near settlements [38,39,40]. These ecological adaptations increase the chances for cross-species transmission and emphasize the pivotal role of wildlife in CPV circulation.

Although the presence of CPV in European wildlife species has been documented in some regions of Italy [21,23,24], Spain [22], and Portugal [19], information on CPV circulation and variant composition among wild animals in Central Europe is still lacking. Here, we address this gap by investigating CPV in two widespread and ecologically important wildlife hosts in Slovakia: the red fox (*Vulpes vulpes*, Canidae) and the European badger (*Meles meles*, Mustelidae). Red foxes may represent a key “bridge” for viral transmission between wildlife reservoirs and domestic animals due to their high population density, ecological adaptability, and frequent contact with domestic dogs [41]. In contrast, the European badger, a widely distributed mustelid closely associated with rural habitats, has been overlooked as a wildlife reservoir of CPV with limited data [21,22]. Recognizing the potential role of these common carnivores is crucial for a more comprehensive understanding of CPV circulation in natural ecosystems and its possible spillover into domestic dog populations.

This study has three main objectives: (i) to investigate the prevalence of CPV strains in red foxes and European badgers in wild habitats across Slovakia; (ii) to identify the circulating CPV variants using molecular characterization combined with virus isolation; and (iii) to elucidate their genetic relationship with strains infecting domestic dogs.

## 2. Materials and Methods

### 2.1. Study Design and Sample Collection

**Figure 1:**
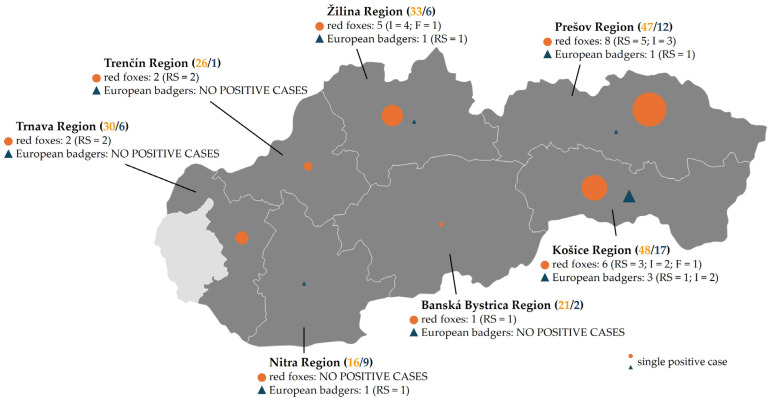
Geographical distribution of CPV positives from wild carnivores in Slovakia. The regions of Slovakia where samples were collected are highlighted. The total number of samples collected from each region is shown in brackets for red foxes (orange) and European badgers (blue), respectively. Positive cases are represented by circles for red foxes (*Vulpes vulpes*) and triangles for European badgers (*Meles meles*). The size of each symbol indicates the number of PCR-positive samples. The number of CPV-positive animals and sample types is mentioned for each region. RS—rectal swab; I—intestine; F—feces. Created with Datawrapper (Datawrapper GmbH, Berlin, Germany; available at: https://www.datawrapper.de/; accessed on 8 August 2025).

This study was conducted in Slovakia during regular hunting seasons (2023–2025), covering seven administrative regions to ensure broad spatial coverage (Figure 1). Sampling sites represented typical rural habitats including forests, meadows, agricultural fields, and peri-urban areas, which are environments commonly inhabited by wild carnivores such as red foxes and European badgers. Most animals included in this study were legally culled by licensed hunters as part of routine wildlife population management and within the framework of passive surveillance for rabies in compliance with Slovak hunting legislation. The hunting season for red foxes is open year-round, whereas for European badgers, it is restricted to 1 September–30 November. No animals were killed specifically for the purposes of this study. All sampling procedures were conducted post-mortem, thereby avoiding any additional stress or harm to the animals. In addition to legally hunted animals, carcasses found dead in the field were also examined.

A total of 209 biological samples (small-intestine samples and rectal swabs) were collected from wild carnivores: red foxes (*n* = 156; 72 intestines, 84 swab samples) and European badgers (*n* = 53; 14 intestines, 39 swabs). Intestinal tissues (*n* = 86) were aseptically collected during post-mortem examination. Rectal swabs (*n* = 123) were used in cases where intestinal tissues were not available (e.g., due to carcass condition or logistical limitations in the field). In addition, freshly deposited or dried fecal samples from red foxes (*n* = 65) were non-invasively collected at locations with confirmed wildlife activity. Sampling sites were selected to minimize spatial overlap while ensuring comprehensive and representative coverage of the study area. All biological samples were collected using sterile techniques, stored in sterile tubes, and transported under the cold chain to the Department of Epizootiology, Parasitology, and Protection of One Health (University of Veterinary Medicine and Pharmacy in Košice), where they were stored at −80 °C until further processing.

### 2.2. DNA Extraction

DNA was extracted from intestinal tissues and rectal swabs using the DNeasy Blood & Tissue Kit and from fecal samples using the QIAamp Fast DNA Stool Mini Kit (both Qiagen, Hilden, Germany), following the manufacturer’s instructions. For each extraction, 25 mg of intestinal tissue or 200 mg of fecal material was used. Rectal swabs were first suspended in 400 µL of phosphate-buffered saline (PBS; pH 7.4), and the resulting suspension was processed. DNA was eluted in 100 µL of elution buffer and stored at −4 °C until analysis. DNA concentration and purity were measured spectrophotometrically (NanoDrop One; Thermo Fisher Scientific, Waltham, MA, USA). A negative extraction control (sterile PBS) was included to monitor potential contamination.

### 2.3. Sample Origin Confirmation

The origin of fox fecal samples was verified using a combination of morphological and molecular approaches. In the field, samples were presumptively identified based on macroscopic morphological characteristics (size, shape, consistency, color, odor, and food residues such as hairs, fruits, or feathers), following the reported criteria for red fox feces [42]. Field identification was performed by experienced hunters familiar with local wildlife species. Species confirmation at the molecular level was conducted by conventional PCR targeting a 503 bp fragment of the mitochondrial cytochrome c oxidase subunit I (COI) gene (corresponding to nucleotides 6228–6730 of the *Vulpes vulpes* reference mitochondrial genome, NC_008434), using species-specific primers (Table 1). PCR was conducted in a 25 µL reaction volume containing 12.5 µL of 2 × PPP Master Mix (Top-Bio, Vestec, Czech Republic), 1 µL of each primer (30 µM), 8.5 µL of PCR-grade water, and 2 µL of DNA template. DNA from the fox intestine served as a positive control. As a no-template negative control, PCR-grade water was included. Cycling conditions were as follows: 94 °C for 1 min; 35 cycles of 94 °C for 15 s, 48 °C for 15 s, 72 °C for 45 s; and final extension at 72 °C for 10 min. Amplicons were stained with GelRed (Biotinum, Fremont, CA, USA), separated by electrophoresis on 1.0% agarose gel in 1 × borax buffer, and visualized with a UVP transilluminator M-15V (Analytik Jena GmbH, Jena, Germany). PCR products were purified using the NucleoSpin Gel and PCR Clean-up Kit Macherey-Nagel GmbH & Co. KG, Düren, Germany) and sequenced using the Sanger method. Sequences were compared to reference sequences deposited in the GenBank database using BLASTn (https://blast.ncbi.nlm.nih.gov/Blast.cgi; accessed on 15 March 2025), with ≥98% identity considered sufficient for species identification [43,44].

### 2.4. Real-Time PCR Screening and DNA Copy Number Determination

Detection of CPV DNA was performed using a probe-based real-time PCR assay targeting a 93 bp fragment of the *VP2* gene (corresponding to nucleotides 4101–4193 of the canine parvovirus 2 reference genome, NC_001539). Primer and probe sequences are listed in Table 1. Reactions were prepared in a total volume of 20 µL using Luna Universal Probe qPCR Master Mix (New England Biolabs, Ipswich, MA, USA), comprising 10 µL of Master Mix, 0.8 µL of each primer (10 µM), 0.4 µL of probe (10 µM), 6 µL of nuclease-free water, and 2 µL of DNA template. Amplification was performed on a qTOWER^3^ real-time PCR system (Analytik Jena GmbH) with the following cycling conditions: 95 °C for 1 min; 40 cycles of 95 °C for 15 s and 55 °C for 30 s. A commercial CPV vaccine (Canigen Puppy 2b; Virbac, Carros, France) and CPV-positive DNA from a previously characterized clinical sample [47] served as positive controls. Each run also included a no-template control (ultrapure water) and a negative extraction control.

CPV DNA copy number in positive samples was relatively quantified using a standard curve generated from the CPV vaccine by plotting Ct values against the logarithm of the copy number. The copy number was estimated from the reference DNA concentration measured spectrophotometrically (NanoDrop One, Thermo Fisher Scientific) and converted to genome copy numbers (copies/µL) using the following formula:

(1) $$copies/µL=\frac{C\times 10^{-9}\times 6.022\times 10^{23}}{\left(N\times MW\right)}$$

Here, C is the DNA concentration (ng/µL), N is the length of the DNA template (CPV genome, ~5200 bp), and MW is the average molecular weight of a nucleotide in ssDNA (330 g/mol). Reference DNA was isolated from the CPV vaccine using the same protocol for field samples to ensure methodological consistency. Briefly, 10-fold serial dilutions (10^8^ to 10^1^ copies/µL) of DNA extracted from the commercial live attenuated CPV vaccine (Canigen Puppy 2b) were prepared. All standard dilutions and samples were tested in triplicate. Assay performance met MIQE-recommended parameters, with amplification efficiency within 90–110% (slope −3.6 to −3.1) and R^2^ ≥ 0.99 [49]. For statistical comparisons of viral DNA loads among sample types or between host species, DNA copy numbers were normalized to the amount of input material.

### 2.5. Conventional PCR Amplification of the VP2 Gene

The full-length *VP2* gene (1755 bp) was amplified from real-time PCR-positive samples in four individual overlapping PCR reactions using specific primer pairs (Table 1). Each reaction was carried out in a total volume of 25 µL containing 12.5 µL TaKaRa Taq HS Perfect Mix (TaKaRa Bio Europe S.A.S., Saint-Germain-en-Laye, France), 0.2 µL of each primer (20 µM), 10.1 µL of PCR-grade water, and 2 µL of DNA template. Cycling conditions were optimized uniformly for all four reactions as follows: 35 cycles of denaturation at 94 °C for 5 s, annealing at 50 °C for 1 s, and extension at 68 °C for 15 s. As a positive control, CPV-positive DNA from a previously characterized clinical sample [47] was used. The resulting amplicons of the expected sizes (554 bp, 541 bp, 563 bp, and 539 bp) were visualized.

### 2.6. Sequencing and Analysis of Amino Acid Residues of the VP2 Protein

PCR amplicons obtained from DNA of the original field samples were purified (NucleoSpin Gel and PCR Clean-up Kit) and Sanger sequenced. For each sample, four overlapping sequence reads were assembled into the full-length *VP2* sequence and aligned using the ClustalW method implemented in Geneious Prime v2025.2.1 (Biomatters Ltd., Auckland, New Zealand). The *VP2* reference sequence (GenBank ID: M38245) was used to ensure correct reading frame alignment. The assembled *VP2* sequences were analyzed with the BLASTn tool and submitted to the GenBank database using the BankIt submission portal.

The *VP2* nucleotide sequences were translated into amino acid sequences to determine the antigenic variant of CPV-2 (2a/2b/2c) based on the amino acid residue at position 426 [50,51]. Translated sequences were compared to the reference strains retrieved from the GenBank database (accessed on 10 August 2025) to examine amino acid substitutions. Sequence analysis and comparative alignments were performed in Geneious Prime v2025.2.1 software.

### 2.7. Phylogenetic Analysis

Phylogenetic analysis was carried out on the *VP2* nucleotide sequences obtained in this study and reference sequences from domestic dogs and wild carnivores retrieved from the GenBank database (accessed on 10 August 2025; Supplementary Materials, Table S1). Multiple-sequence alignment was performed using the ClustalW algorithm in MEGA v12 software (Molecular Evolutionary Genetics Analysis, Temple University, Philadelphia, PA, USA) [52]. The best-fit substitution model was selected based on the Bayesian Information Criterion (BIC). The phylogenetic tree was constructed using a maximum likelihood statistical method using the Tamura-3 parameter model (T92) with discrete Gamma distribution (five rate categories) (G) and invariant sites (I). Node support was assessed with 1000 bootstrap replicates. Bootstrap values ≥ 50% were indicated at corresponding nodes.

### 2.8. Cell Culture and Virus Isolation

To isolate the virus, CPV-positive samples (intestine, rectal swabs, and fecal material) were processed accordingly and inoculated on the Madin-Darby Canine Kidney (MDCK; ATCC, Manassas, VA, USA) cell line.

Briefly, MDCK cells were grown to confluence in Minimal Essential Medium (MEM; Sigma-Aldrich, Darmstadt, Germany) supplemented with 10% fetal bovine serum (FBS) (Sigma-Aldrich) and 1% antibiotic antimycotic solution (10,000 U/mL penicillin, 10 mg/mL streptomycin, and 25 µg/mL amphotericin B; Sigma-Aldrich) at 37 °C with 5% CO_2_ with 95% humidity. The samples were homogenized (10% *w*/*v*) in serum-free MEM (Sigma–Aldrich) supplemented with 1% antibiotic and antimycotic solution (Sigma-Aldrich). Suspensions were clarified by centrifugation (8000× *g*, 20 min, room temperature), and the resulting supernatants were filtered through 0.22 µm syringe filters (TPP Techno Plastic Products AG, Trasadingen, Switzerland) to remove cellular debris and potential bacterial or fungal contamination. The homogenates were serially diluted (10^−1^–10^−4^) in a serum-free MEM medium supplemented with 1% antibiotic and antimycotic solution (Sigma-Aldrich). One milliliter of diluted homogenate was added to the MDCK monolayer (~90% confluency) in a T25 tissue culture flask (TPP). Non-infected cells were used as a negative control. For positive control, viral supernatant from the CPV-positive dog was used [47]. Inoculated MDCK cells were incubated at 37 °C in a 5% CO_2_ incubator. After 1 h of adsorption, the inoculum was removed and fresh 4 mL of MEM supplemented with 2% FBS (Sigma-Aldrich) and 1% antibiotic antimycotic solution (Sigma-Aldrich) was added. Cells were cultured at 37 °C in 5% CO_2_ for 5–6 days and monitored daily using an inverted microscope (Optica S.r.l., Ponteranica, Italy). Viral growth was evaluated by detection of the cytopathic effect (CPE). When no CPE was observed, blind passages were performed. Three consecutive passages were performed to test viral replication. Monolayers were subjected to three cycles of freezing and thawing, and the collected lysates were centrifuge (1500× *g*, 10 min, 4 °C). The supernatants were subsequently tested for the presence of CPV DNA by PCR targeting the *VP2* gene (primers are listed in Table 1).

### 2.9. Statistical Analysis

CPV positivity rates were calculated with 95% confidence intervals using the Wilson/Brown method. Viral DNA copy numbers were first tested for normality using the Shapiro–Wilk test. As the data did not follow a normal distribution, the nonparametric test Mann–Whitney U test was applied. Differences in prevalence between sample types (intestines, rectal swabs, feces) were assessed using the Chi-square test and pairwise Fisher’s exact tests. Statistical significance was set at *p* < 0.05. Statistical analyses were performed using GraphPad Prism v8.0 (GraphPad Software, San Diego, CA, USA).

## 3. Results

### 3.1. Study Area and Distribution of Samples

During the study period, 156 red foxes were culled, comprising 82 males (52.6%) and 74 females (47.4%), all classified as adults. For adult European badgers (*n* = 53), sex data were not available. Fecal samples (*n* = 65) from red foxes were collected non-invasively as part of pathogen surveillance (Table 2). Samples were obtained from seven of the eight administrative regions of Slovakia (Figure 1). The Bratislava Region was not represented due to the absence of suitable specimens during the study period. Intestinal samples and rectal swabs from red foxes were collected from all represented regions. In European badgers, sampling coverage varied by region: only rectal swabs were available from the Žilina and Banská Bystrica regions, whereas only intestinal tissue was available from the Trenčín region (Table 3). The highest number of red fox samples (48/221; 21.7%) and European badger samples (17/53; 32.1%) originated from the Košice Region (Figure 1 and Table 3).

### 3.2. Fecal Samples Originate from Red Foxes

To confirm the species origin of non-invasively collected fecal samples, a two-step approach was employed. A total of 65 fecal samples were initially identified in situ based on the established identification criteria (Supplementary Materials, Figure S1) [42]. To minimize the risk of species misidentification in areas where sympatric carnivores co-occur, molecular confirmation of host species was carried out. A 503 bp fragment of the mitochondrial COI gene, a widely used DNA barcode for vertebrate species identification, was successfully amplified in all field samples (Supplementary Materials, Figure S2). Sanger sequencing revealed identical sequences among all amplicons. BLAST analysis showed a high sequence identity (98.01–100%) with *V. vulpes* reference sequences deposited in GenBank. Additional matches corresponded to other *Vulpes* spp. with < 98% identity. No matches were found with non-fox or non-canid species (Supplementary Materials, Table S2). Taken together, the molecular confirmation fully supported the field-based morphological assessment, and no discrepancies between these two approaches were observed.

### 3.3. Screening for CPV

Real-time PCR screening revealed the presence of CPV DNA in 30 out of 274 tested specimens (10.9%; 95% CI: 7.8–15.2), with 17/30 positive rectal swabs, 11/30 intestines, and 2/65 fecal samples. Among red fox samples, the overall prevalence was 10.9% (24/221; 95% CI: 7.4–15.6). Positive results were found in all three sample types: rectal swabs (13/84; 15.5%; 95% CI: 9.3–24.7), intestinal tissues (9/72; 12.5%; 95% CI: 6.7–22.1), and fecal samples (2/65; 3.1%; 95% CI: 0.5–10.5). In 11.3% positive European badgers (6/53; 95% CI: 5.3–22.6), CPV DNA was detected in 4 out of 39 rectal swabs (10.3%; 95% CI: 4.1–23.6) and 2/14 intestinal tissues (14.3%; 95% CI: 2.5–39.9) (Table 2). No overall difference in CPV prevalence among different sampling sources (intestines, rectal swabs, and feces) was observed (Chi-square test, *p* = 0.065). Fecal samples had a significantly lower prevalence (2/65; 3.1%) compared to intestinal tissues (11/86; 12.8%; pairwise Fisher’s exact test, *p* = 0.042) and rectal swabs (17/123; 13.8%, *p* = 0.021), while no difference was observed between intestines and swabs (*p* = 1.000).

The highest regional CPV prevalence was detected in samples collected from the Žilina Region (6/39; 15.4%; 95% CI: 7.3–29.7), while the lowest rates were recorded in the Nitra Region (1/25; 4%; 95% CI: 0.2–19.5) and the Banská Bystrica Region (1/23; 4.3%; 95% CI: 0.2–21) with a single positive case. Among red foxes, the highest CPV prevalence was observed in the Prešov Region (8/47; 17%; 95% CI: 8.9–30.1), whereas the positivity rate among European badgers was highest in the Košice Region (3/17; 17.6%; 95% CI: 6.2–41). No positives were detected from red foxes in Nitra Region, while badgers from the Trenčín and the Trnava Regions were tested negative. The detailed distribution of CPV-positive samples according to the sampled region and animal species is presented in Table 3. Given unequal sampling across regions, these regional differences should be interpreted cautiously.

The overall median copy number of CPV DNA in positive samples was 2.05 × 10^3^ (range: 8.7 × 10^1^–5.73 × 10^5^), with 9.75 × 10^3^ copies/mg in intestinal tissues, 4.54 × 10^2^ copies/µL in rectal swabs, and 4.27 × 10^2^ copies/mg in feces. CPV DNA copy numbers did not differ between the rectal swabs of foxes (median: 4.54 × 10^2^ copies/µL) and badgers (median: 4.75 × 10^2^ copies/µL) (Mann–Whitney U, *p* = 0.6235). In contrast, intestinal tissues from badgers exhibited significantly higher DNA copy numbers (median: 3.67 × 10^5^ copies/mg) compared to foxes (median: 8.74 × 10^3^ copies/mg) (Mann–Whitney U, *p* = 0.0364). Fecal samples, available only from red foxes, were excluded from pairwise statistical analysis. Details are presented in Table 4 and Supplementary Materials, Table S3.

### 3.4. VP2 Sequence and Phylogenetic Analysis

The full-length *VP2* gene was successfully amplified by conventional PCR and directly sequenced from all 30 CPV-positive samples. Nucleotide sequences of wild CPV strains identified in this study were deposited in GenBank under accession numbers PX146837–PX146866 (Table 5).

According to the analysis of the *VP2* gene sequences obtained from viral screening, 20 out of 30 isolates (66.7%) were classified as CPV-2b, 9 isolates (30%) as CPV-2a, and one isolate (3.3%) as CPV-2c based on the VP2 426—Asp (D), Asn (N), and Glu (E) residues, respectively. A similar distribution of antigenic variants was observed across host species. Among red foxes (*n* = 24), CPV-2b was detected in sixteen sequences, CPV-2a in seven sequences, and one isolate belonged to CPV-2c. In European badgers (*n* = 6), CPV-2b was identified in four isolates and CPV-2a in two isolates (Table 4).

The sequences from this study showed 98.80–100% nucleotide identity and 98.63–100% amino acid identity (Supplementary Materials, Figure S3). The sequence matrix identity of the identified VP2 sequences and reference strains revealed high similarity within each genotype at both nucleotide (99.26–100%) and amino acid levels (99.32–100%). Details on genotype-specific comparisons are provided in Supplementary Materials, Figure S4.

**Figure 2:**
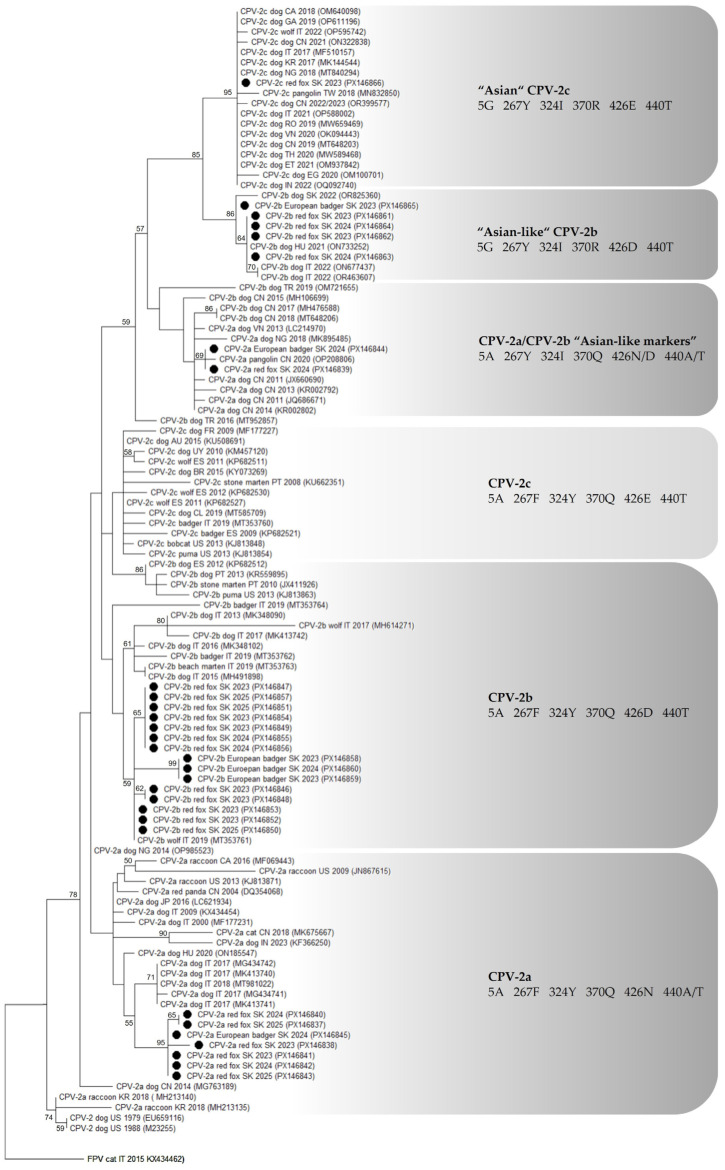
Phylogenetic analysis of CPV-2 isolates detected in this study. The phylogenetic tree was constructed based on the complete *VP2* nucleotide sequences (1755 nt) of CPV-2 strains obtained in this study and reference strains in the GenBank database (Supplementary Materials, Table S1). Feline panleukopenia virus (FPV; GenBank accession no. KX434462) was used as an out-group. The phylogenetic tree was constructed by MEGA v12 using the maximum likelihood statistical method and the Tamura-3 parameter model (T92) with discrete gamma distribution (five rate categories) (G) and invariant sites (I). Statistical support was provided by bootstrapping with 1000 replicates. The black circles represent the sequences identified in this study. Bootstrap values ≥ 50% are indicated at the respective branches. The antigenic variants and selected amino acid residues in positions 5, 267, 324, 370, 426, and 440 are reported.

The VP2 amino acid sequences of isolates from this study were compared with the reference sequences of CPV-2 strains detected in dogs and wild carnivores. Emphasis was placed on residues at positions 5, 267, 324, 370, and 440, which have been highlighted in the literature as sites of relevant substitutions contributing to CPV-2 genetic variability (Table 5). Within the CPV-2a antigenic variants (*n* = 9), five sequences obtained from red foxes (sample IDs: RF-F-58, RF-I-40, RF-RS-28, RF-RS-51, and RF-RS-82; Acc. nos.: PX146837, PX146840–PX146843, respectively) and one sequence from European badger (sample ID: EB-RS-29; Acc. no.: PX146845) shared an identical amino acid profile (5A, 267F, 297A, 324Y, 370Q, and 440T). One CPV-2a sequence from red fox (sample ID: RF-I-17; Acc. no.: PX146838) differed from this group by a single non-synonymous mutation, Ile447Met (I447M). Phylogenetic analysis confirmed that these Slovak isolates clustered within the CPV-2a lineage, together with reference strains from domestic dogs and wild carnivores in Europe (Hungary and Italy), indicating their genetic relatedness to circulating European CPV-2a strains (Figure 2). Two CPV-2a sequences, one from red fox (sample ID: RF-I-32; Acc. no.: PX146839) and one from European badger (sample ID: EB-I-11; Acc. no.: PX146844), were identical to each other and differed from the above-mentioned group at two substitutions: Phe267Tyr (F267Y) and Tyr324Ile (Y324I). Phylogenetic analysis revealed that these CPV-2a sequences were closely related to some CPV-2a strains bearing some (i.e., 267Y, 324I) of the typical “Asian-like” signature (5G, 267Y, 324I, 370R). Accordingly, we designate them as CPV-2a carrying “Asian-like markers” (Figure 2). Among the CPV-2b variants (*n* = 20), twelve sequences obtained from red foxes (sample IDs: RF-I-6, RF-I-12, RF-I-15, RF-I-27, RF-I-64, RF-I-66, RF-RS-2, RF-RS-7, RF-RS-14, RF-RS-39, RF-RS-47, and RF-RS-64; Acc. nos.: PX146846–PX146857) displayed an identical amino acid pattern at the examined positions (5A, 267F, 297A, 324Y, and 370Q). Three sequences from European badgers (sample IDs: EB-I-2, EB-RS-13, and EB-RS-31; Acc. nos.: PX146858–PX146860) exhibited the same profile, except for a single Ser552Ile (S552I) substitution. Phylogenetic analysis placed these Slovak CPV-2b isolates within the CPV-2b clade, clustering together with reference strains from Italy, indicating their close genetic relatedness to previously reported dog- and wildlife-associated CPV-2b strains in Europe (Figure 2). In contrast, a subset of five CPV-2b sequences, four from red foxes and one from European badgers (sample IDs: RF-F-26, RF-RS-5, RF-RS-32, RF-RS-73, and EB-RS-8; Acc. no.: PX146861–PX146865), demonstrated a different amino acid signature characterized by substitutions at residues Ala5Gly, Phe267Tyr, Tyr324Ile, and Gln370Arg. In the phylogenetic analysis, these CPV-2b strains clustered together with CPV-2b isolates detected in dogs from Italy, Hungary, and Slovakia in recent years, known as “Asian-like” CPV-2b based on “Asian-like” signature (5G, 267Y, 324I, and 370R) (Figure 2). The single CPV-2c VP2 sequence obtained from the red fox (sample ID: RF-RS-23; Acc. no.: PX146866) exhibited a distinct amino acid composition defined by substitutions Ala5Gly, Phe267Tyr, Tyr324Ile, and Asn370Arg in comparison with reference CPV-2c strains (Table 5). This specific combination of amino acids was previously reported in CPV-2c isolates from Asia, Europe, and Africa, which cluster phylogenetically within the clade commonly referred to as “Asian” CPV-2c. Consistent with this molecular signature, the Slovak isolate was placed within the same clade, clustering together with reference strains of the “Asian” CPV-2c lineage (Figure 2). Details of the amino acid composition of each isolate from this study are presented in Supplementary Materials, Table S4.

### 3.5. Virus Isolation on MDCK Cell Line

**Figure 3:**
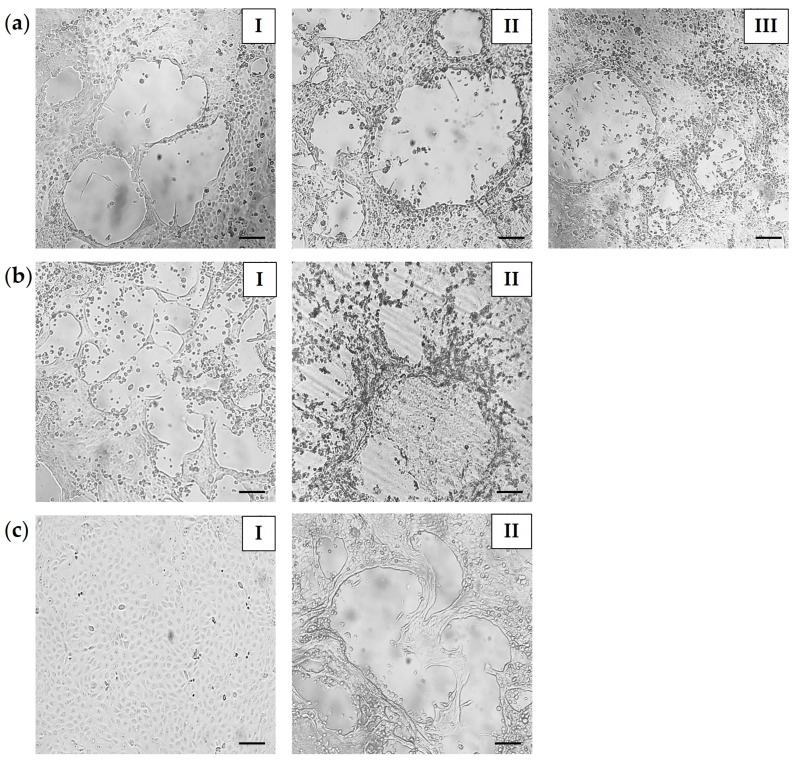
Cytopathic effect of representative CPV isolates identified in this study on MDCK cells. (**a**) red fox samples: (**I**) intestine (sample ID: RF-I-17; PX146838), (**II**) rectal swab (sample ID: RF-RS-28; PX146841), (**III**) feces (sample ID: RF-F-26; PX146861); (**b**) European badger samples: (**I**) intestine (sample ID: EB-I-11; PX146844), (**II**) rectal swab (sample ID: EB-RS-31; PX146860); (**c**) controls: (**I**) negative control—non-infected MDCK; (**II**) positive control—viral supernatant from CPV-positive dog (OR825360) [47]. Scale bars = 100 µm.

Isolation of CPV was performed by inoculating MDCK cell monolayers with suspensions prepared from tissue, swab, and fecal samples. Viral growth was assessed based on the development of CPE. The inoculation of undiluted homogenates frequently resulted in rapid destruction of the cell monolayer within 24 h–48 h. This effect was considered nonspecific, and most likely related to cytotoxic components present in the samples. In contrast, inoculation with serially diluted suspensions (10^−1^–10^−2^) reduced non-specific cytotoxic effects, enabling the detection of characteristic parvovirus-associated CPE, consisting of cell rounding, plaque formation, clumping, and the detachment of infected cells from the monolayer (Figure 3). The onset of CPE was influenced by the nature of the inoculated material. Tissue-derived suspensions induced characteristic morphological alterations in cell cultures in the first passage (4–5 days post-infection, dpi). Swab specimens usually required one to two passages before CPE became evident. In contrast, fecal suspensions produced detectable CPE only after the second or third blind passage. Details of CPE appearance are presented in Supplementary Materials, Table S5. After three consecutive passages, the presence of CPV in the harvested cultures showing CPE was confirmed by the amplification of the 573 bp fragment of the *VP2* gene (Supplementary Materials, Figure S5).

## 4. Discussion

Wild animals play a pivotal role in the ecology of pathogens traditionally associated with domestic animals [33,38]. Canine parvovirus (CPV), endemic in dog populations, has also been reported in a wide range of wild carnivores [19,20,21,22,23,24,25,32]. Nevertheless, many aspects of CPV infection in wildlife, including its epidemiological dynamics, remain unclear [53]. Although transmission between domestic and wild carnivores has been reported, the direction of this spillover and the role of wildlife in long-term virus persistence are still not well understood [19,31,33]. The CPV occurrence in wildlife has often been regarded as spillovers from domestic animals, particularly in peri-urban areas where domestic-wildlife interactions are common [28,53,54]. However, recent evidence indicates that wild carnivores may also act as reservoirs, facilitating spillover into other susceptible species, including unvaccinated domestic dogs or vulnerable wild canids [37,55]. Field studies are needed to resolve infection dynamics, host range, and cross-species transmission, especially for synanthropic species, contributing to CPV maintenance and transmission. Clarifying their role will help prioritize surveillance and refine conservation and veterinary management.

In this study, we investigated the role of red foxes and European badgers as potential wildlife hosts of CPV-2 in Slovakia. These species were selected as representatives of two widespread and ecologically important carnivore families (Canidae and Mustelidae) frequently occurring in wildlife–domestic contact zones. By combining molecular analysis and virus isolation, we aimed to provide new insights into the diversity of CPV circulating among wild carnivores in Slovakia.

The geographical coverage of our sampling encompassed seven out of the eight regions of Slovakia, providing a broad overview of CPV occurrence in the country. Sample numbers were uneven, reflecting a sampling strategy linked to routine wildlife population management and passive rabies surveillance. A similar limitation of geographical representation has been reported in studies from other European countries, where sample availability was often constrained by hunting or death from vehicular trauma [19,21,22]. Still, the dataset encompassed both western and eastern parts of the country, which is essential as regional differences in host density, human population pressure, and contact rates between wildlife and domestic carnivores may influence CPV transmission dynamics.

In contrast to previous studies that analyzed tissue samples such as spleen, liver, or intestinal segments [21,22,23,24,27], our study employed a multi-matrix sampling strategy, encompassing intestinal tissue, rectal swabs, and fecal samples. The intestinal tissue has been demonstrated as the most reliable tissue sample to collect from wild animals for parvovirus detection, with a 54% detection rate, compared to only 2.8% in spleen samples [21]. Rectal swabs and feces enable minimally invasive or non-invasive sampling, which is particularly advantageous in wildlife surveillance. This diversified approach significantly enhances the CPV detection probability among variable field-derived specimens, expanding diagnostic sensitivity. The sample dataset, comprising adult individuals because of the culling practices, may increase the probability of CPV detection due to their longer environmental exposure and higher potential for subclinical infections. Overall, the spatial coverage and sample size (221 red foxes and 53 European badgers) provide a valuable baseline for monitoring CPV in wild carnivores in Slovakia.

Species assignment of fecal samples is a critical step in non-invasive pathogen surveillance, as morphological identification can be error-prone, particularly in regions where sympatric carnivores produce similar fecal characteristics. By combining field-based criteria [42] with COI DNA barcoding, we confirmed the fecal samples’ attribution to red foxes. Although similarity with other *Vulpes* species was shown, pairwise identity was below the cutoff of 98% applied for species confirmation [43,44]. Moreover, only *V. vulpes* is known to be present in Slovakia (according to Act No. 274/2009 Coll. on Hunting, National Council of the Slovak Republic). Thus, sequence similarity with other closely related *Vulpes* species is biologically irrelevant. This dual approach eliminated potential cross-species misclassification, which has been identified as a limitation in previous wildlife studies [56,57]. Monterroso and colleagues documented misclassification rates of ~14% for red fox feces and even higher error levels for other carnivores when only morphological traits were used [56].

In this study, a total prevalence of 10.9% was observed for CPV in wild carnivore species, consistent with the 1–18% range from other European countries [19,21,22,23,24], but contrasts with the higher rates (55.9%) reported by Duarte et al. [58]. For red foxes, 10.9% of samples were tested positive for CPV. This prevalence is comparable to findings from Northern Italy (15.9%) [24] and exceeds the lower rates commonly observed in other wildlife studies from Spain (2.4%) [22] or Turkey (0.3%) [25]. In contrast, Duarte et al. reported a substantially higher prevalence in foxes (78.9%) [58]. At the same time, several studies failed to detect CPV in any of the red foxes examined [19,21,23]. Such variability may reflect ecological and epidemiological factors, including host density, viral persistence in the environment, and cross-species transmission. In badgers, CPV was detected in 11.3% of tested samples. To date, molecular evidence of CPV infection in badgers remains limited. Previous studies reported either high CPV prevalence in a small sample set from Italy (6/10, 60%) [21], relatively low prevalence in Spain (8/68, 8.8%) [22], or the complete absence in tested badger populations [19,23,27]. Comparable prevalence in both host species suggests that badgers may also be exposed to CPV, although their precise role in virus maintenance remains to be clarified.

CPV prevalence varied regionally, with the highest virus circulation in the Žilina Region (15.4%) and lowest in the Nitra Region (4%). Foxes showed the most positives in the Prešov Region (17%) and badgers in the Košice Region (17.6%). However, the obtained results may have been biased by the non-homogenous sampling in the regions. For instance, the number of sampled badgers from the Trenčín and the Banská Bystrica Region was too low (*n* = 1 and *n* = 2, respectively) to claim that the CPV is not present there in these animals. The unbalanced sample sizes also restrict interspecies comparisons.

Positive results were obtained from all three sample types, supporting the value of a multi-sample approach for CPV surveillance in wildlife. Rectal swabs showed a considerable proportion of positive detections (56.7%; 17/30). This supports their diagnostic value as a minimally invasive sampling method. Positive intestinal samples (36.7%, 11/30), tended to demonstrate higher viral loads (9.75 × 10^3^ copies/mg), a trend consistent with the known intestinal tropism of CPV [59] and earlier reports from wild carnivores [21]. Badger intestines showed significantly higher values (3.67 × 10^5^ DNA copies/mg) compared to foxes (8.74 × 10^3^ DNA copies/mg), although this finding should be interpreted with caution, given the limited number of positive badger samples (*n* = 4). However, these findings raise the possibility that badgers may support higher viral burdens, a hypothesis that requires further investigation in larger datasets. Fecal material has often been overlooked in CPV surveillance of wildlife, with most studies focusing exclusively on tissues collected post-mortem [19,21,22,23,24]. This underrepresentation may lead to an incomplete picture of viral shedding and transmission pathways. From an epidemiological perspective, feces remain highly relevant, since they represent the main route of CPV shedding and environmental contamination. Although the positivity rate in fecal samples was lower in our study (3.1%), this finding is consistent with reports from Turkey, with overall CPV prevalence 0.3% in 630 examined fox feces [25]. The relatively low DNA copy numbers detected in fecal samples (4.27 × 10^2^ DNA copies/mg) may be explained by the limited number of positives (*n* = 2) together with the well-known variability of viral shedding, the presence of PCR inhibitors [60,61,62], and the generally lower viral quantities in this matrix compared to internal organs [63,64]. Low-viral DNA quantities in feces are also a well-recognized limitation in CPV diagnostics and represent a major reason for false-negative results in antigen-based assays [65]. Nevertheless, detection of CPV DNA in feces remains a key indicator of active virus excretion into the environment and should be considered a complementary tool for large-scale, non-invasive monitoring.

Overall, CPV-2b was the predominant parvovirus variant detected in this study (66.7%), consistent with reports from other countries [19,21,24,25]. CPV-2a was identified in one-third of cases, confirming its ongoing circulation in Europe. In contrast, CPV-2c was detected only once in our dataset. However, this variant has also been documented in several other European wildlife studies, indicating that, although less common, CPV-2c is circulating among domestic dog populations and is capable of infecting wild carnivore hosts [19,21,22,23]. Our results demonstrate the co-circulation of multiple antigenic variants in Central Europe, consistent with the complex CPV epidemiology previously described in wild hosts across the continent [19,21,22,24].

CPV sequences obtained in this study carried alanine (A) at amino acid position 297 of the VP2 protein that defines the so-called “new” variants. The Ser297Ala substitution has been circulating predominantly among domestic dogs worldwide since the 1990s and is suspected to have replaced the original variant [14]. This mutation is considered critical in host–pathogen interactions and is likely to arise as a result of host adaptation of the virus [66,67]. Moreover, it has been associated with positive selection and may contribute to immune escape [66]. All *VP2* sequences analyzed in this study also encoded glycine (G) at position 300. This residue is highly conserved among most CPV-2 variants and has been shown to be essential for infection of canine hosts [68,69].

Variability among our isolates was primarily observed at residues 5, 267, 324, and 370, which are recognized as key residues of CPV-2 molecular evolution. Mutations at these sites occur in regions of high antigenicity or in domains involved in transferrin receptor binding, thereby contributing to viral adaptation, influencing the host range, and to the emergence of novel vaccine escape mutants [14,15,66]. Most CPV-2a (PX146837–PX146838, PX146840–PX146843, PX146845) and CPV-2b isolates (PX146846–PX146860) displayed the unchanged amino acid profile (5A, 267F, 324Y, 370Q) and clustered phylogenetically with European reference strains from domestic dogs and wild carnivores (Hungary, Italy), indicating that the viruses circulating within fox and badger populations in Slovakia are part of the CPV lineage distributed in Europe and maintained through interspecies transmission [21,70,71]. One mutation, Ile447Met, was observed in a single CPV-2a isolate (PX146838). This substitution has also been reported in CPV-2 strains from domestic dogs [72], although its significance remains unclear. Since residue 447 is not exposed on the capsid surface, the changes at this site may not directly affect viral antigenicity. However, possible conformational effects on capsid structure cannot be excluded [72]. Three CPV-2b isolates from European badgers (PX146858–PX146860) retained the typical amino acid profile (5A, 267F, 324Y, 370Q), but displayed an additional substitution, Ser552Ile. Residue 552 lies within the C-terminal region of the VP2, distant from the virus–receptor interaction zone, but encompassing antigenic sites exposed to the host immune system [69]. Mutations in this region have been documented to affect antigenicity in related parvoviruses [73]. To the best of our knowledge, the S552I substitution has not been reported previously in CPV, and its functional significance is presently unknown. While speculative, this mutation may influence the VP2 structure that modulates antibody recognition. The five CPV-2b sequences (PX146861–PX146865) carried the amino acid substitutions Ala5Gly, Phe267Tyr, Tyr324Ile, and Gln370Arg, markers characteristic for “Asian” CPV-2c strains (5G, 267Y, 324I, 370R), and typically referred to as “Asian-like” CPV-2b [74]. Thr440Ala is also regarded as an “Asian-like” marker and, together with Phe267Tyr and Tyr324Ile, has been suggested to represent vaccine escape mutants [14]. In the present study, these three mutations were not simultaneously observed. The sequences retained threonine at position 440, contrasting with recent isolates from domestic or wild animals [14,16,24,75]. These CPV-2b isolates clustered with CPV-2b strains known as “Asian-like” CPV-2b strains reported recently in European domestic dogs from Italy [74], Hungary [76], and Slovakia [47]. Considering that this variant has already been confirmed in our previous study [47], these findings strongly suggest that “Asian-like” CPV-2b is now circulating locally, with cross-species transmission. This may reflect recurrent spillover at the wildlife–domestic interface rather than the persistence of wildlife-restricted lineages. Two CPV-2a sequences (PX146839 and PX146844) displayed two of the typical “Asian-like” amino acid mutation patterns, Phe267Tyr and Tyr324Ile. Phylogenetically, these isolates clustered with reference sequences carrying a partial “Asian-like” signature. For the purposes of this study, and to distinguish them from “Asian-like”’ strains, we therefore designated them as CPV-2a with “Asian-like markers”. Magliocca et al. documented a red fox isolate carrying the 324I substitution [24], indicating that CPV-2a variants with partial “Asian-like” signatures have been detected sporadically in European wildlife populations. The amino acid profile (5G, 267Y, 324I, 370R) clustered the CPV-2c isolate (PX146866) within the “Asian” CPV-2c lineage, together with isolates from Asia, Africa, and Europe. In Europe, “Asian” CPV-2c has been reported in domestic dogs from Italy [23] or Romania [70] and in a wolves from Italy [23].

Phylogenetic analysis revealed that Slovak CPV-2a and CPV-2b strains from wildlife clustered closely with European domestic dog strains (mainly Italy and Hungary), which is consistent with previous observations from other countries [25,77,78]. This pattern is generally interpreted as evidence of spillover from domestic dogs, which represent the main reservoir with high prevalence, into wild carnivores [37,55]. Several sequences carried amino acid signatures characteristic of “Asian” or “Asian-like” variants (5G, 267Y, 324I, 370R. Interestingly, all these CPV-2 isolates were detected in eastern Slovakia. This geographical pattern may reflect introduction through transboundary movement, a factor previously implicated in the spread of “Asian-like” CPV variants in Europe [16], followed by spillover into local sympatric wildlife populations. Taken together, our data suggest that CPV circulation in wildlife largely reflects the viral diversity found in domestic dogs. While the phylogenetic signal supports domestic-to-wild spillover as the most likely scenario, the exact direction of transmission at the domestic–wildlife interface remains uncertain, and bidirectional exchange between host species cannot be excluded.

In contrast to previous wildlife studies, which relied on molecular detection, this work also demonstrates successful virus isolation, confirming viable CPV in wildlife hosts. Characteristic CPV-associated CPE (plaque-forming and partial lysis of the MDCK monolayer) was observed for all positive sample types. Virus isolation from fecal specimens is particularly challenging due to the presence of cytotoxic or inhibitory compounds. Kurucay et al. (2023) reported the successful isolation of CPV-2b from red fox feces in one out of two positive samples [25], reflecting the limited efficiency of virus recovery from this matrix. Nevertheless, the successful isolation of CPV from both positive fecal materials in our study highlights its epidemiological relevance, confirming that feces can contain viable, infectious virus particles capable of contaminating the environment and contributing to CPV transmission.

To the best of our knowledge, this study provides the first evidence of CPV in Central European wildlife, confirmed by both molecular characterization and virus isolation. Our findings expand current knowledge on the genetic diversity of CPV circulating in wild carnivores in Europe and highlight the role of wildlife in CPV epidemiology. Results from this study may contribute to the increased need for integrated surveillance and control strategies across domestic and wildlife hosts.

This study has some limitations. First, the sample size was restricted to animals collected from a single country. Although seven out of the eight administrative regions of Slovakia were included, the geographic coverage is still limited and may not fully represent CPV diversity across Central Europe. Second, the number of European badgers analyzed was considerably lower compared to red foxes, which may have biased prevalence estimates for this host species. Third, only the *VP2* gene was analyzed, while whole-genome data would provide a more comprehensive view of viral evolution and recombination. Fourth, the number of fecal samples was limited and is available only for foxes, which may have influenced prevalence estimates by sample type. Finally, the cross-sectional nature of the sampling does not allow us to assess temporal dynamics of CPV circulation. Despite these limitations, our findings provide novel insights into CPV prevalence and genetic diversity in wildlife hosts in Central Europe.

## 5. Conclusions

This study provides the first evidence of CPV-2 circulation in Central European wildlife, confirmed by both molecular detection and virus isolation. The identification of CPV-2a, CPV-2b, and CPV-2c variants, including “Asian-like” lineages, demonstrates the genetic diversity of circulating CPV strains and suggests that wildlife may contribute to the maintenance of these globally emerging viruses. Our findings underscore the ongoing risk of cross-species transmission and pathogen spillover between domestic animals and wildlife. Sustained molecular surveillance and strengthened collaboration between veterinary and wildlife sectors will be crucial to monitor viral evolution and mitigate risks to both domestic and wild carnivore populations.

## Figures and Tables

**Table 1:** Primers used in this study.

Primer/Probe	Sequence (5′–3′)	Position	Amplicon Size	Reference
Sample-origin confirmation				
VV-COI-F	CACTGTAGGAATAGATGTGG	6228–6247 ^a^	503 bp	this study
VV-COI-R	AGATGAATGAGCCTATAGATG	6710–6730 ^a^		
CPV screening				
qPCR-CPV-F	AAACAGGAATTAACTATACTAATATATTTA	4101–4130 ^b^	93 bp	[45]
qPCR-CPV-R	AAATTTGACCATTTGGATAAACT	4171–4193 ^b^		
qPCR-CPV-P	FAM–TGGTCCTTTAACTGCATTAAATAATGTACC-BHQ1	4138–4167 ^b^		
VP2 sequencing				
PCR I				
VP2-F ^c^	AGAGACAATCTTGCACCAAT	2768–2787 ^b^	554 bp	[46]
CPV-VP2-INT-R1	CTATCTAATGCAACCATCAATG	3300–3321 ^b^		[47]
PCR II				
CPV-VP2-INT-F1	GTTGCATTTAGTTAGTTTTGAACA	3190–3213 ^b^	541 bp	[47]
CPV-VP2-INT-R2	ACCACGTCTTTTATCTTGTTG	3710–3730 ^b^		
PCR III				
CPV-VP2-INT-F2	GATTGTAAACCATGTAGACTAACA	3590–3613 ^b^	563 bp	[47]
CPV-VP2-INT-R3	GCAGTTAAAGGACCATAAGTA	4132–4152 ^b^		
PCR IV				
CPV-VP2-INF-F3	GAAGATATCCAGAAGGAGATTGG	4005–4027 ^b^	539 bp	[47]
VP2-R ^d^	ATGTTAATATAATTTTCTAGGTGCT	4519–4543 ^b^		[46]
Confirmation of virus isolation				
degCPV-VP2-F	TGATGGAGSAGTWCAACCAGA	2791–2811 ^b^	573 bp	[47]
CPV-VP2-R	TCAGATCTCATAGCTGCTGGA	3343–3363 ^b^		[48]

^a^ Primer positions are referred to the reference sequence of *Vulpes vulpes* mitochondrion complete genome (accession no.: NC_008434). ^b^ Primer positions are referred to the reference sequence of the canine parvovirus 2 complete genome (accession no.: NC_001539). ^c^ Primer comprises the 3′ end part of the *VP1* gene (18 bp). ^d^ Primer comprises the initial part (3 bp) of the 3′ untranslated region (3′UTR) of canine parvovirus genome.

**Table 2:** Sample collection summary.

Host Species	No. of Samples		Sample Type		Total Positives	Prevalence (95% CI)
		Rectal Swabs Pos./Total (%)	Intestines Pos./Total (%)	Feces Pos./Total (%)		
red fox	221	13/84 (15.5%)	9/72 (12.5%)	2/65 (3.1%)	24/221	10.9% (7.4–15.6)
European badger	53	4/39 (10.3%)	2/14 (14.3%)	–	6/53	11.3% (5.3–22.6)
Total	274	17/123 (13.8%)	11/86 (12.8%)	2/65 (3.1%)	30/274	10.9% (7.8–15.2)

Please note that fecal samples were collected only from red foxes and are therefore indicated as “–” for European badgers.

**Table 3:** Details on sample collection summary by region and species.

Region	Host Species	No. of Samples	Sample Type			Total CPV Positives	Positivity Rate
			Rectal Swabs Pos./Total (%)	Intestines Pos./Total (%)	Feces Pos./Total (%)		
Banská Bystrica	Red fox	21	1/9	0/5	0/7	1/21	4.8%
	European badger	2	0/2	N/A	–	0/2	–
	Total	23	1/11	0/5	0/7	1/23	4.3%
Košice	Red fox	48	3/15	2/19	1/14	6/48	12.5%
	European badger	17	1/12	2/5	–	3/17	17.6%
	Total	65	4/27	4/24	1/14	9/65	13.8%
Nitra	Red fox	16	0/7	0/4	0/5	0/16	–
	European badger	9	1/7	0/2	–	1/9	11.1%
	Total	25	1/14	0/6	0/5	1/25	4%
Prešov	Red fox	47	5/21	3/17	0/9	8/47	17%
	European badger	12	1/9	0/3	–	1/12	8.3%
	Total	59	6/30	3/20	0/9	9/59	15.2%
Trenčín	Red fox	26	2/11	0/7	0/8	2/26	7.7%
	European badger	1	N/A	0/1	–	0/1	0
	Total	27	2/11	0/8	0/8	2/27	7.4%
Trnava	Red fox	30	2/10	0/8	0/12	2/30	6.7%
	European badger	6	0/3	0/3	–	0/6	–
	Total	36	2/13	0/11	0/12	2/36	5.5%
Žilina	Red fox	33	0/11	4/12	1/10	5/33	15.1%
	European badger	6	1/6	N/A	–	1/6	16.6%
	Total	39	1/17	4/12	1/10	6/39	15.4%

N/A—sample type not available. Please note that fecal samples were collected only from red foxes and are therefore indicated as “–” for European badgers.

**Table 4:** Details of samples from red foxes and European badgers that tested positive for CPV.

Region	Host Species	Tested Sample	Sample ID	Mean Ct	DNA Copy Number/ µL Template	DNA Copy Number/ Input Material *	CPV-2 Variant
Banská Bystrica Region	red fox	rectal swab	RF-RS-7	27.92	1.47 × 10^3^	3.68 × 10^2^	CPV-2b
Košice Region	red fox	rectal swab	RF-RS-5	28.01	1.38 × 10^3^	3.45 × 10^2^	CPV-2b
	red fox	rectal swab	RF-RS-32	27.62	1.81 × 10^3^	4.54 × 10^2^	CPV-2b
	red fox	rectal swab	RF-RS-64	28.27	1.15 × 10^3^	2.88 × 10^2^	CPV-2b
	red fox	intestine	RF-I-12	27.2	2.44 × 10^3^	9.75 × 10^3^	CPV-2b
	red fox	intestine	RF-I-27	27.64	1.79 × 10^3^	7.15 × 10^3^	CPV-2b
	red fox	feces	RF-F-26	29.61	4.47 × 10^2^	2.23 × 10^2^	CPV-2b
	European badger	rectal swab	EB-RS-8	28.39	1.05 × 10^3^	2.64 × 10^2^	CPV-2b
	European badger	intestine	EB-I-2	21.42	1.43 × 10^5^	5.73 × 10^5^	CPV-2b
	European badger	intestine	EB-I-11	23.22	4.02 × 10^4^	1.61 × 10^4^	CPV-2a
Nitra Region	European badger	rectal swab	EB-RS-29	25.22	9.86 × 10^3^	2.47 × 10^3^	CPV-2a
Prešov Region	red fox	rectal swab	RF-RS-14	27.48	2 × 10^3^	5 × 10^2^	CPV-2b
	red fox	rectal swab	RF-RS-23	28.26	1.16 × 10^3^	2.9 × 10^2^	CPV-2c
	red fox	rectal swab	RF-RS-39	28.7	8.51 × 10^2^	2.13 × 10^2^	CPV-2b
	red fox	rectal swab	RF-RS-47	27.35	2.19 × 10^3^	5.48 × 10^2^	CPV-2b
	red fox	rectal swab	RF-RS-73	28.28	1.14 × 10^3^	2.85 × 10^2^	CPV-2b
	red fox	intestine	RF-I-17	29.4	5.2 × 10^2^	2.08 × 10^3^	CPV-2b
	red fox	intestine	RF-I-32	28.94	7.19 × 10^2^	2.88 × 10^3^	CPV-2a
	red fox	intestine	RF-I-64	24.61	1.52 × 10^4^	6.06 × 10^4^	CPV-2b
	European badger	rectal swab	EB-RS-13	29.97	3.48 × 10^2^	8.7 × 10^1^	CPV-2b
Trenčín Region	red fox	rectal swab	RF-RS-2	25.5	8.10 × 10^3^	2.02 × 10^3^	CPV-2b
	red fox	rectal swab	RF-RS-51	24.46	1.68 × 10^4^	4.21 × 10^3^	CPV-2a
Trnava Region	red fox	rectal swab	RF-RS-28	25.3	9.29 × 10^3^	2.32 × 10^3^	CPV-2a
	red fox	rectal swab	RF-RS-82	23.8	2.68 × 10^4^	6.7 × 10^3^	CPV-2a
Žilina Region	red fox	intestine	RF-I-6	27.39	2.13 × 10^3^	8.53 × 10^3^	CPV-2b
	red fox	intestine	RF-I-15	26.81	3.21 × 10^3^	1.28 × 10^4^	CPV-2b
	red fox	intestine	RF-I-40	27.36	2.19 × 10^3^	8.74 × 10^3^	CPV-2a
	red fox	intestine	RF-I-66	25.18	1.01 × 10^4^	4.06 × 10^4^	CPV-2b
	red fox	feces	RF-F-58	28.14	1.26 × 10^3^	6.31 × 10^2^	CPV-2a
	European badger	rectal swab	EB-RS-31	27.04	2.74 × 10^3^	6.85 × 10^2^	CPV-2b

* Please note that DNA copy number was calculated per defined input sample amounts (i.e., copies/mg for intestine and fecal samples or copies/µL for rectal swabs).

**Table 5:** Amino acid variations in the VP2 sequence of the wild CPV-2 isolates analyzed in this study and reference CPV-2 strains.

CPV-2 Variant	Host	Country	Year	Accession No.		Amino Acid Residues								
					5	267	297	300	324	370	426	440	447	552
CPV-2	dog	USA	1979	EU659116	A	F	S	A	Y	Q	N	T	I	S
CPV-2a	dog	Italy	2009	KX434454	A	F	A	G	Y	Q	N	A	I	S
	raccoon	Canada	2016	MF069443	A	F	A	D	Y	Q	N	T	I	S
	**red fox ^1^**	**Slovakia**	**2023–2025**	**PX146837, PX146840–PX146843**	**A**	**F**	**A**	**G**	**Y**	**Q**	**N**	**T**	**I**	**S**
	**red fox**	**Slovakia**	**2023**	**PX146838**	**A**	**F**	**A**	**G**	**Y**	**Q**	**N**	**T**	**M**	**S**
	**E. badger ^1^**	**Slovakia**	**2024**	**PX146845**	**A**	**F**	**A**	**G**	**Y**	**Q**	**N**	**T**	**I**	**S**
CPV-2a “Asian-like markers”	dog	China	2011	JX660690	A	Y	A	G	I	Q	N	A	I	S
	red fox	Italy	2022	PP551646	-	-	A	G	I	Q	N	A	I	-
	**red fox**	**Slovakia**	**2024**	**PX146839**	**A**	**Y**	**A**	**G**	**I**	**Q**	**N**	**T**	**I**	**S**
	**E. badger**	**Slovakia**	**2024**	**PX146844**	**A**	**Y**	**A**	**G**	**I**	**Q**	**N**	**T**	**I**	**S**
CPV-2b	dog	Portugal	2013	KR559895	A	F	A	G	Y	Q	D	T	I	S
	badger	Italy	2019	MT353762	A	F	A	G	Y	Q	D	T	I	S
	**red fox ^2^**	**Slovakia**	**2023–2025**	**PX146846–PX146857**	**A**	**F**	**A**	**G**	**Y**	**Q**	**D**	**T**	**I**	**S**
	**E. badger ^3^**	**Slovakia**	**2023–2024**	**PX146858–PX146860**	**A**	**F**	**A**	**G**	**Y**	**Q**	**D**	**T**	**I**	**I**
“Asian-like” CPV-2b	dog	Italy	2022	ON677437	G	Y	A	G	I	R	D	T	I	S
	**red fox ^4^**	**Slovakia**	**2023–2024**	**PX146861–PX146864**	**G**	**Y**	**A**	**G**	**I**	**R**	**D**	**T**	**I**	**S**
	**E. badger ^4^**	**Slovakia**	**2023**	**PX146865**	**G**	**Y**	**A**	**G**	**I**	**R**	**D**	**T**	**I**	**S**
CPV-2c	dog	Italy	2000	FJ005195	A	F	A	G	Y	Q	E	T	I	S
	E. badger	Spain	2012	KP682530	A	F	A	G	Y	Q	E	T	I	S
“Asian” CPV-2c	dog	Italy	2022	OR463608	G	Y	A	G	I	R	E	T	I	S
	pangolin	Taiwan	2018	MN832850	G	Y	A	G	I	R	E	T	I	S
	wolf	Italy	2022	OP595742	G	Y	A	G	I	R	E	T	I	S
	**red fox**	**Slovakia**	**2023**	**PX146866**	**G**	**Y**	**A**	**G**	**I**	**R**	**E**	**T**	**I**	**S**

^1^ VP2 amino acid sequences of identified CPV-2a isolates from both wild animals were identical. ^2^ VP2 amino acid sequences of identified CPV-2b isolates from red foxes were identical. ^3^ VP2 amino acid sequences of identified CPV-2b isolates from European badgers were identical. ^4^ VP2 amino acid sequences of identified “Asian-like” CPV-2b isolates from both wild animals were identical. The sequences generated in this study are displayed in bold. VP2 amino acid numbering follows the start of the VP2 ORF (aa 1–584). Abbreviations: A—alanine (Ala), D—aspartic acid (Asp), E—glutamic acid (Glu), F—phenylalanine (Phe), G—glycine (Gly), I—isoleucine (Ile), M—methionine (Met), Q—glutamine (Gln), R—arginine (Arg), S—serine (Ser), T—threonine (Thr), Y—tyrosine (Tyr), “–” information is not available.

## Data Availability

The original data presented in the study are openly available in GenBank public database under accession numbers PX146837–PX146866.

## Supplementary Materials

The following supporting information can be downloaded at: https://www.mdpi.com/article/10.3390/microorganisms13102325/s1, Figure S1: Fecal samples from red foxes (*Vulpes vulpes*) collected in the field; Figure S2: Representative gel electrophoresis of COI PCR products (503 bp) amplified from red fox fecal DNA; Figure S3: Similarity identity matrix of the *VP2* nucleotide sequences (lower triangle, highlighted in green/yellow) and amino acid sequences (upper triangle, highlighted in red/blue) of the CPV-2 isolates from this study; Figure S4: Similarity identity matrix of the *VP2* nucleotide sequences (highlighted in green/yellow, lower triangle) and amino acid sequences (highlighted in red/blue, upper triangle) of the CPV-2 isolates from this study and reference strains; Figure S5: Confirmation of CPV isolation on MDCK cell line; Table S1: List of reference sequences used in this study for phylogenetic analysis; Table S2: List of sequences producing significant alignments with the partial *COI* gene sequence from fecal samples using the NCBI BLASTn algorithm; Table S3: Statistical analysis of CPV DNA copy numbers normalized to input material by host and sample type; Table S4: Amino acid and nucleotide profile observed in CPV-2 sequences from wild carnivores identified in this study; Table S5: Summary of CPE appearance in MDCK cell cultures for each CPV-positive sample.

## Abbreviations

The following abbreviations are used in this manuscript:

CPE	cytopathic effect
CPV	canine parvovirus
EB	European badger
F	fecal sample
I	small intestine
MDCK	Madin-Darby Canine Kidney
MEM	Minimal Essential Medium
N/A	not available
RF	red fox
RS	rectal swab
