# Supplementary material for: Red Foxes (Vulpes vulpes) and European Badgers (Meles meles) as Overlooked Wildlife Hosts of Canine Parvovirus in Slovakia: First Evidence by Molecular Characterization and Virus Isolation
Source: Microorganisms. 2025 Oct 8;13(10):2325. doi: 10.3390/microorganisms13102325 (PMC12566571; doi:10.3390/microorganisms13102325)
Supplement: Supplementary file 1 [file microorganisms-13-02325-s001.zip › microorganisms-3897904-supplementary.pdf]

Red Foxes (*Vulpes vulpes*) and European Badgers (*Meles meles*) as Overlooked Wildlife Hosts of Canine Parvovirus in Slovakia: First Evidence by Molecular Characterization and Virus Isolation

Patrícia Petroušková [1,] *, Andrea Pelegrinová [1], Jozef Lazár [2], Jakub Lipinský [1], Monika Drážovská [1], Marián Prokeš [1], Ľuboš Korytár [1], Boris Vojtek [1], Maroš Kostičák [1], Ladislav Molnár [3], Jana Mojžišová Vaščinec [1] and Anna Ondrejková [1]

[1] Department of Epizootiology, Parasitology and Protection of One Health, University of Veterinary Medicine and Pharmacy in Košice, Komenského 73, 041 81, Košice, Slovakia
[2] Department of Breeding and Diseases of Game, Fish and Bees, Ecology and Cynology, University of Veterinary Medicine and Pharmacy in Košice, Komenského 73, 041 81, Košice, Slovakia
[3] Clinic of Birds, Exotic and Free Living Animals, University of Veterinary Medicine and Pharmacy in Košice, Komenského 73, 041 81, Košice, Slovakia
* Correspondence: patricia.petrouskova@uvlf.sk

Supplementary Figures

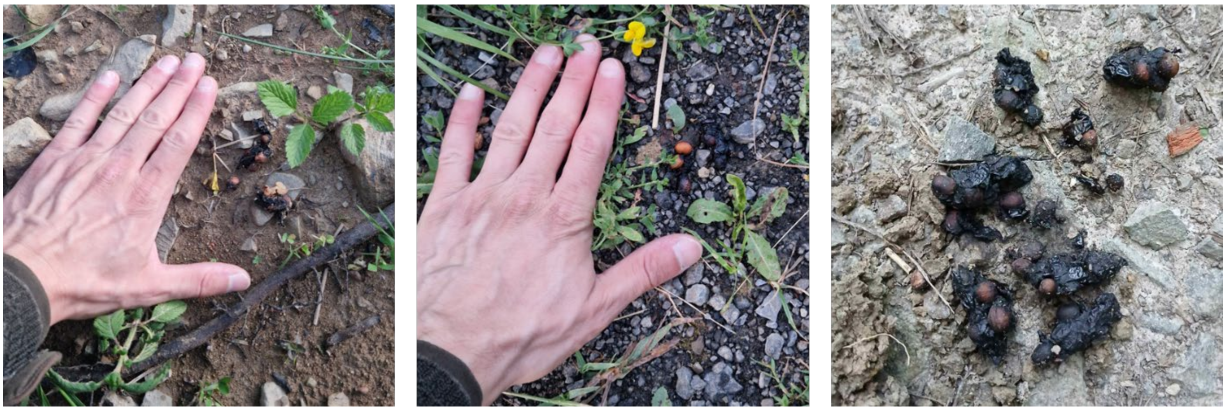

Figure S1. Fecal samples from red foxes (*Vulpes vulpes*) collected in the field. Feces were morphologically identified as originating from red foxes (*Vulpes vulpes*) based on characteristic macroscopic features such as shape, size, consistency, color, odor, and visible dietary remains (e.g., hairs, feathers, fruits). Identification was performed by trained hunters familiar with local wildlife.

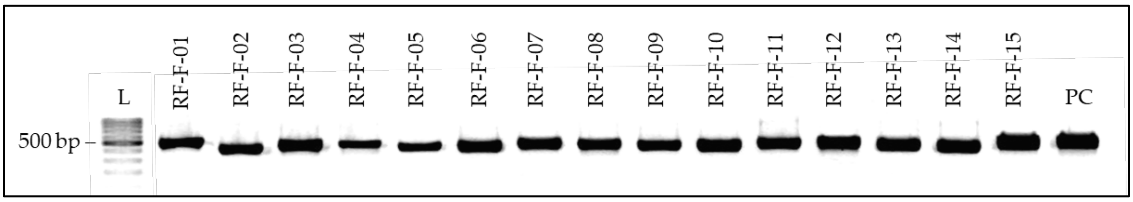

Figure S2. Representative gel electrophoresis of *COI* PCR products (503 bp) amplified from red fox fecal DNA. L – 100 bp DNA Ladder; RF-F-01 – 15: representative sample amplicons; PC – positive control (fox intestinal DNA).

	CPV-2a (PX146837)	CPV-2a (PX146838)	CPV-2a (PX146840)	CPV-2a (PX146841)	CPV-2a (PX146842)	CPV-2a (PX146843)	CPV-2a (PX146845)	CPV-2a "Asian-like markers" (PX146839)	CPV-2a "Asian-like markers" (PX146844)	CPV-2b (PX146846)	CPV-2b (PX146847)	CPV-2b (PX146848)	CPV-2b (PX146849)	CPV-2b (PX146850)	CPV-2b (PX146851)	CPV-2b (PX146852)	CPV-2b (PX146853)	CPV-2b (PX146854)	CPV-2b (PX146855)	CPV-2b (PX146856)	CPV-2b (PX146857)	CPV-2b (PX146858)	CPV-2b (PX146859)	CPV-2b (PX146860)	"Asian-like" CPV-2b (PX146861)	"Asian-like" CPV-2b (PX146862)	"Asian-like" CPV-2b (PX146863)	"Asian-like" CPV-2b (PX146864)	"Asian-like" CPV-2b (PX146865)	"Asian" CPV-2c (PX146866)
CPV-2a (PX146837)	ID	99.8	100	100	100	100	100	99.66	99.66	99.49	99.49	99.49	99.49	99.49	99.49	99.49	99.49	99.49	99.49	99.49	99.49	99.32	99.32	99.32	99.14	99.14	99.14	99.14	99.14	99.1
CPV-2a (PX146838)	99.83	ID	99.83	99.83	99.83	99.83	99.83	99.49	99.49	99.32	99.32	99.32	99.32	99.32	99.32	99.32	99.32	99.32	99.32	99.32	99.32	99.14	99.14	99.14	98.97	98.97	98.97	98.97	98.97	99
CPV-2a (PX146840)	100	99.8	ID	100	100	100	100	99.66	99.66	99.49	99.49	99.49	99.49	99.49	99.49	99.49	99.49	99.49	99.49	99.49	99.49	99.32	99.32	99.32	99.14	99.14	99.14	99.14	99.14	99.1
CPV-2a (PX146841)	99.94	99.9	99.94	ID	100	100	100	99.66	99.66	99.49	99.49	99.49	99.49	99.49	99.49	99.49	99.49	99.49	99.49	99.49	99.49	99.32	99.32	99.32	99.14	99.14	99.14	99.14	99.14	99.1
CPV-2a (PX146842)	99.94	99.9	99.94	100	ID	100	100	99.66	99.66	99.49	99.49	99.49	99.49	99.49	99.49	99.49	99.49	99.49	99.49	99.49	99.49	99.32	99.32	99.32	99.14	99.14	99.14	99.14	99.14	99.1
CPV-2a (PX146843)	99.94	99.9	99.94	100	100	ID	100	99.66	99.66	99.49	99.49	99.49	99.49	99.49	99.49	99.49	99.49	99.49	99.49	99.49	99.49	99.32	99.32	99.32	99.14	99.14	99.14	99.14	99.14	99.1
CPV-2a (PX146845)	99.94	99.9	99.94	100	100	100	ID	99.66	99.66	99.49	99.49	99.49	99.49	99.49	99.49	99.49	99.49	99.49	99.49	99.49	99.49	99.32	99.32	99.32	99.14	99.14	99.14	99.14	99.14	99.1
CPV-2a "Asian-like markers" (PX146839)	99.43	99.4	99.43	99.49	99.49	99.49	99.49	ID	100	99.14	99.14	99.14	99.14	99.14	99.14	99.14	99.14	99.14	99.14	99.14	99.14	98.97	98.97	98.97	99.49	99.49	99.49	99.49	99.49	99.5
CPV-2a "Asian-like markers" (PX146844)	99.43	99.4	99.43	99.49	99.49	99.49	99.49	100	ID	99.14	99.14	99.14	99.14	99.14	99.14	99.14	99.14	99.14	99.14	99.14	99.14	98.97	98.97	98.97	99.49	99.49	99.49	99.49	99.49	99.5
CPV-2b (PX146846)	99.26	99.2	99.26	99.32	99.32	99.32	99.32	99.37	99.37	ID	100	100	100	100	100	100	100	100	100	100	100	99.83	99.83	99.83	98.97	98.97	98.97	98.97	98.97	98.8
CPV-2b (PX146847)	99.26	99.2	99.26	99.32	99.32	99.32	99.32	99.26	99.26	99.89	ID	100	100	100	100	100	100	100	100	100	100	99.83	99.83	99.83	98.97	98.97	98.97	98.97	98.97	98.8
CPV-2b (PX146848)	99.26	99.2	99.26	99.32	99.32	99.32	99.32	99.37	99.37	100	99.89	ID	100	100	100	100	100	100	100	100	100	99.83	99.83	99.83	98.97	98.97	98.97	98.97	98.97	98.8
CPV-2b (PX146849)	99.26	99.2	99.26	99.32	99.32	99.32	99.32	99.26	99.26	99.89	100	99.89	ID	100	100	100	100	100	100	100	100	99.83	99.83	99.83	98.97	98.97	98.97	98.97	98.97	98.8
CPV-2b (PX146850)	99.32	99.3	99.32	99.37	99.37	99.37	99.37	99.32	99.32	99.94	99.94	99.94	99.94	ID	100	100	100	100	100	100	100	99.83	99.83	99.83	98.97	98.97	98.97	98.97	98.97	98.8
CPV-2b (PX146851)	99.26	99.2	99.26	99.32	99.32	99.32	99.32	99.26	99.26	99.89	100	99.89	100	99.94	ID	100	100	100	100	100	100	99.83	99.83	99.83	98.97	98.97	98.97	98.97	98.97	98.8
CPV-2b (PX146852)	99.32	99.3	99.32	99.37	99.37	99.37	99.37	99.32	99.32	99.94	99.94	99.94	99.94	100	99.94	ID	100	100	100	100	100	99.83	99.83	99.83	98.97	98.97	98.97	98.97	98.97	98.8
CPV-2b (PX146853)	99.32	99.3	99.32	99.37	99.37	99.37	99.37	99.32	99.32	99.94	99.94	99.94	99.94	100	99.94	100	ID	100	100	100	100	99.83	99.83	99.83	98.97	98.97	98.97	98.97	98.97	98.8
CPV-2b (PX146854)	99.26	99.2	99.26	99.32	99.32	99.32	99.32	99.26	99.26	99.89	100	99.89	100	99.94	100	99.94	99.94	ID	100	100	100	99.83	99.83	99.83	98.97	98.97	98.97	98.97	98.97	98.8
CPV-2b (PX146855)	99.26	99.2	99.26	99.32	99.32	99.32	99.32	99.26	99.26	99.89	100	99.89	100	99.94	100	99.94	99.94	100	ID	100	100	99.83	99.83	99.83	98.97	98.97	98.97	98.97	98.97	98.8
CPV-2b (PX146856)	99.26	99.2	99.26	99.32	99.32	99.32	99.32	99.26	99.26	99.89	100	99.89	100	99.94	100	99.94	99.94	100	100	ID	100	99.83	99.83	99.83	98.97	98.97	98.97	98.97	98.97	98.8
CPV-2b (PX146857)	99.26	99.2	99.26	99.32	99.32	99.32	99.32	99.26	99.26	99.89	100	99.89	100	99.94	100	99.94	99.94	100	100	100	ID	99.83	99.83	99.83	98.97	98.97	98.97	98.97	98.97	98.8
CPV-2b (PX146858)	99.09	99.2	99.09	99.15	99.15	99.15	99.15	99.09	99.09	99.72	99.72	99.72	99.72	99.77	99.72	99.77	99.77	99.72	99.72	99.72	99.72	ID	100	100	98.8	98.8	98.8	98.8	98.8	98.6
CPV-2b (PX146859)	99.09	99.2	99.09	99.15	99.15	99.15	99.15	99.09	99.09	99.72	99.72	99.72	99.72	99.77	99.72	99.77	99.77	99.72	99.72	99.72	99.72	100	ID	100	98.8	98.8	98.8	98.8	98.8	98.6
CPV-2b (PX146860)	99.09	99.2	99.09	99.15	99.15	99.15	99.15	99.09	99.09	99.72	99.72	99.72	99.72	99.77	99.72	99.77	99.77	99.72	99.72	99.72	99.72	100	100	ID	98.8	98.8	98.8	98.8	98.8	98.6
"Asian-like" CPV-2b (PX146861)	98.97	98.8	98.97	98.92	98.92	98.92	98.92	99.32	99.32	99.15	99.15	99.15	99.15	99.09	99.15	99.09	99.09	99.15	99.15	99.15	99.15	99.09	99.09	99.09	ID	100	100	100	100	99.8
"Asian-like" CPV-2b (PX146862)	98.97	98.8	98.97	98.92	98.92	98.92	98.92	99.32	99.32	99.15	99.15	99.15	99.15	99.09	99.15	99.09	99.09	99.15	99.15	99.15	99.15	99.09	99.09	99.09	100	ID	100	100	100	99.8
"Asian-like" CPV-2b (PX146863)	98.97	98.8	98.97	98.92	98.92	98.92	98.92	99.32	99.32	99.15	99.15	99.15	99.15	99.09	99.15	99.09	99.09	99.15	99.15	99.15	99.15	99.09	99.09	99.09	100	100	ID	100	100	99.8
"Asian-like" CPV-2b (PX146864)	98.97	98.8	98.97	98.92	98.92	98.92	98.92	99.32	99.32	99.15	99.15	99.15	99.15	99.09	99.15	99.09	99.09	99.15	99.15	99.15	99.15	99.09	99.09	99.09	100	100	100	ID	100	99.8
"Asian-like" CPV-2b (PX146865)	98.92	98.9	98.92	98.97	98.97	98.97	98.97	99.37	99.37	99.2	99.2	99.2	99.2	99.15	99.2	99.15	99.15	99.2	99.2	99.2	99.2	99.15	99.15	99.15	99.94	99.94	99.94	99.94	ID	99.8
"Asian" CPV-2c (PX146866)	98.86	98.8	98.86	98.92	98.92	98.92	98.92	99.32	99.32	99.15	99.15	99.15	99.15	99.09	99.15	99.09	99.09	99.15	99.15	99.15	99.15	98.97	98.97	98.97	99.66	99.66	99.66	99.66	99.72	ID

Sequence matrix identity (%)

98 98.2 98.4 98.6 98.8 99 99.2 99.4 99.6 99.8 100

98 98.2 98.4 98.6 98.8 99 99.2 99.4 99.6 99.8 100

Figure S3. Similarity identity matrix of the *VP2* nucleotide sequences (lower triangle, highlighted in green/yellow) and amino acid sequences (upper triangle, highlighted in red/blue) of the CPV-2 isolates from this study. The sequences from this study showed 98.80% – 100% nucleotide identity and 98.63% – 100% amino acid identity. CPV-2a sequences showed 99.89% – 100% nucleotide and amino acid identity. CPV-2a with "Asian-like markers" sequences displayed 100% identity. CPV-2b isolates 99.72% – 100% nucleotide and 99.83% – 100% amino acid identity. "Asian-like" CPV-2b showed 99.94% – 100% nucleotide and complete (100%) amino acid identity.

	CPV-2 (EU659116)	CPV-2a (MK413741)	CPV-2a (MT981022)	CPV-2a (MF069443)	CPV-2a (KX434454)	**CPV-2a (PX146837)**	**CPV-2a (PX146838)**	**CPV-2a (PX146840)**	**CPV-2a (PX146841)**	**CPV-2a (PX146842)**	**CPV-2a (PX146843)**	**CPV-2a (PX146845)**	CPV-2a "Asian-like markers" (JX660690)	CPV-2a "Asian-like markers" (JQ686671)	CPV-2a "Asian-like markers" (KR002792)	CPV-2a "Asian-like markers" (LC214970)	**CPV-2a "Asian-like markers" (PX146839)**	**CPV-2a "Asian-like markers" (PX146844)**	CPV-2b (KR559895)	CPV-2b (MT353761)	CPV-2b (MT353762)	CPV-2b (MT353763)	**CPV-2b (PX146846)**	**CPV-2b (PX146847)**	**CPV-2b (PX146848)**	**CPV-2b (PX146849)**	**CPV-2b (PX146850)**	**CPV-2b (PX146851)**	**CPV-2b (PX146852)**	**CPV-2b (PX146853)**	**CPV-2b (PX146854)**	**CPV-2b (PX146855)**	**CPV-2b (PX146856)**	**CPV-2b (PX146857)**	**CPV-2b (PX146858)**	**CPV-2b (PX146859)**	**CPV-2b (PX146860)**	"Asian-like" CPV-2b (ON733252)	"Asian-like" CPV-2b (OR825360)	"Asinal-like" CPV-2b (ON677437)	"Asian-like" CPV-2b (OR463607)	**"Asian-like" CPV-2b (PX146861)**	**"Asian-like" CPV-2b (PX146862)**	**"Asian-like" CPV-2b (PX146863)**	**"Asian-like" CPV-2b (PX146864)**	**"Asian-like" CPV-2b (PX146865)**	CPV-2c (FJ005195)	CPV-2c (KP682511)	CPV-2c (KP682530)	"Asian" CPV-2c (OP595742)	"Asian" CPV-2c (MN832850)	"Asian" CPV-2c (MW659469)	"Asian" CPV-2c (OR463608)	**"Asian" CPV-2c (PX146866)**
CPV-2 (EU659116)	ID	98.80	98.80	98.80	98.80	98.97	98.80	98.97	98.97	98.97	98.97	98.97	98.29	98.46	98.46	98.46	98.63	98.63	98.80	98.46	98.46	98.46	98.46	98.46	98.46	98.46	98.46	98.46	98.46	98.46	98.46	98.46	98.46	98.46	98.29	98.29	98.29	98.12	98.12	98.12	98.12	98.12	98.12	98.12	98.12	98.12	98.63	98.80	98.63	97.95	98.12	98.12	98.12	98.12
CPV-2a (MK413741)	99.32	ID	100.00	99.32	99.66	99.83	99.66	99.83	99.83	99.83	99.83	99.83	99.32	99.49	99.49	99.49	99.66	99.66	99.66	99.32	99.32	99.32	99.32	99.32	99.32	99.32	99.32	99.32	99.32	99.32	99.32	99.32	99.32	99.32	99.14	99.14	99.14	99.14	99.14	99.14	99.14	99.14	99.14	99.14	99.14	99.14	99.49	99.66	99.49	98.97	99.14	99.14	99.14	99.14
CPV-2a (MT981022)	99.32	100.00	ID	99.32	99.66	99.83	99.66	99.83	99.83	99.83	99.83	99.83	99.32	99.49	99.49	99.49	99.66	99.66	99.66	99.32	99.32	99.32	99.32	99.32	99.32	99.32	99.32	99.32	99.32	99.32	99.32	99.32	99.32	99.32	99.14	99.14	99.14	99.14	99.14	99.14	99.14	99.14	99.14	99.14	99.14	99.14	99.49	99.66	99.49	98.97	99.14	99.14	99.14	99.14
CPV-2a (MF069443)	99.43	99.54	99.54	ID	99.32	99.49	99.32	99.49	99.49	99.49	99.49	99.49	98.80	98.97	98.97	98.97	99.14	99.14	99.32	98.97	98.97	98.97	98.97	98.97	98.97	98.97	98.97	98.97	98.97	98.97	98.97	98.97	98.97	98.97	98.80	98.80	98.80	98.63	98.63	98.63	98.63	98.63	98.63	98.63	98.63	98.63	99.14	99.32	99.14	98.46	98.63	98.63	98.63	98.63
CPV-2a (KX434454)	99.49	99.72	99.72	99.72	ID	99.83	99.66	99.83	99.83	99.83	99.83	99.83	99.49	99.66	99.66	99.66	99.49	99.49	99.66	99.32	99.32	99.32	99.32	99.32	99.32	99.32	99.32	99.32	99.32	99.32	99.32	99.32	99.32	99.32	99.14	99.14	99.14	98.97	98.97	98.97	98.97	98.97	98.97	98.97	98.97	98.97	99.49	99.66	99.49	98.80	98.97	98.97	98.97	98.97
**CPV-2a (PX146837)**	99.20	99.66	99.66	99.43	99.60	ID	99.83	100.00	100.00	100.00	100.00	100.00	99.32	99.49	99.49	99.49	99.66	99.66	99.83	99.49	99.49	99.49	99.49	99.49	99.49	99.49	99.49	99.49	99.49	99.49	99.49	99.49	99.49	99.49	99.32	99.32	99.32	99.14	99.14	99.14	99.14	99.14	99.14	99.14	99.14	99.14	99.66	99.83	99.66	98.97	99.14	99.14	99.14	99.14
**CPV-2a (PX146838)**	99.15	99.60	99.60	99.37	99.54	99.83	ID	99.83	99.83	99.83	99.83	99.83	99.14	99.32	99.32	99.32	99.49	99.49	99.66	99.32	99.32	99.32	99.32	99.32	99.32	99.32	99.32	99.32	99.32	99.32	99.32	99.32	99.32	99.32	99.14	99.14	99.14	98.97	98.97	98.97	98.97	98.97	98.97	98.97	98.97	98.97	99.49	99.66	99.49	98.80	98.97	98.97	98.97	98.97
**CPV-2a (PX146840)**	99.20	99.66	99.66	99.43	99.60	100.00	99.83	ID	100.00	100.00	100.00	100.00	99.32	99.49	99.49	99.49	99.66	99.66	99.83	99.49	99.49	99.49	99.49	99.49	99.49	99.49	99.49	99.49	99.49	99.49	99.49	99.49	99.49	99.49	99.32	99.32	99.32	99.14	99.14	99.14	99.14	99.14	99.14	99.14	99.14	99.14	99.66	99.83	99.66	98.97	99.14	99.14	99.14	99.14
**CPV-2a (PX146841)**	99.26	99.72	99.72	99.49	99.66	99.94	99.89	99.94	ID	100.00	100.00	100.00	99.32	99.49	99.49	99.49	99.66	99.66	99.83	99.49	99.49	99.49	99.49	99.49	99.49	99.49	99.49	99.49	99.49	99.49	99.49	99.49	99.49	99.49	99.32	99.32	99.32	99.14	99.14	99.14	99.14	99.14	99.14	99.14	99.14	99.14	99.66	99.83	99.66	98.97	99.14	99.14	99.14	99.14
**CPV-2a (PX146842)**	99.26	99.72	99.72	99.49	99.66	99.94	99.89	99.94	100.00	ID	100.00	100.00	99.32	99.49	99.49	99.49	99.66	99.66	99.83	99.49	99.49	99.49	99.49	99.49	99.49	99.49	99.49	99.49	99.49	99.49	99.49	99.49	99.49	99.49	99.32	99.32	99.32	99.14	99.14	99.14	99.14	99.14	99.14	99.14	99.14	99.14	99.66	99.83	99.66	98.97	99.14	99.14	99.14	99.14
**CPV-2a (PX146843)**	99.26	99.72	99.72	99.49	99.66	99.94	99.89	99.94	100.00	100.00	ID	100.00	99.32	99.49	99.49	99.49	99.66	99.66	99.83	99.49	99.49	99.49	99.49	99.49	99.49	99.49	99.49	99.49	99.49	99.49	99.49	99.49	99.49	99.49	99.32	99.32	99.32	99.14	99.14	99.14	99.14	99.14	99.14	99.14	99.14	99.14	99.66	99.83	99.66	98.97	99.14	99.14	99.14	99.14
**CPV-2a (PX146845)**	99.26	99.72	99.72	99.49	99.66	99.94	99.89	99.94	100.00	100.00	100.00	ID	99.32	99.49	99.49	99.49	99.66	99.66	99.83	99.49	99.49	99.49	99.49	99.49	99.49	99.49	99.49	99.49	99.49	99.49	99.49	99.49	99.49	99.49	99.32	99.32	99.32	99.14	99.14	99.14	99.14	99.14	99.14	99.14	99.14	99.14	99.66	99.83	99.66	98.97	99.14	99.14	99.14	99.14
CPV-2a "Asian-like markers" (JX660690)	99.09	99.49	99.49	99.32	99.60	99.32	99.26	99.32	99.37	99.37	99.37	99.37	ID	99.83	99.83	99.83	99.66	99.66	99.14	98.80	98.80	98.80	98.80	98.80	98.80	98.80	98.80	98.80	98.80	98.80	98.80	98.80	98.80	98.80	98.63	98.63	98.63	99.14	99.14	99.14	99.14	99.14	99.14	99.14	99.14	99.14	98.97	99.14	98.97	98.97	99.14	99.14	99.14	99.14
CPV-2a "Asian-like markers" (JQ686671)	99.09	99.49	99.49	99.32	99.60	99.32	99.26	99.32	99.37	99.37	99.37	99.37	99.89	ID	100.00	100.00	99.83	99.83	99.32	98.97	98.97	98.97	98.97	98.97	98.97	98.97	98.97	98.97	98.97	98.97	98.97	98.97	98.97	98.97	98.80	98.80	98.80	99.32	99.32	99.32	99.32	99.32	99.32	99.32	99.32	99.32	99.14	99.32	99.14	99.14	99.32	99.32	99.32	99.32
CPV-2a "Asian-like markers" (KR002792)	99.03	99.43	99.43	99.26	99.54	99.26	99.20	99.26	99.32	99.32	99.32	99.32	99.83	99.83	ID	100.00	99.83	99.83	99.32	98.97	98.97	98.97	98.97	98.97	98.97	98.97	98.97	98.97	98.97	98.97	98.97	98.97	98.97	98.97	98.80	98.80	98.80	99.32	99.32	99.32	99.32	99.32	99.32	99.32	99.32	99.32	99.14	99.32	99.14	99.14	99.32	99.32	99.32	99.32
CPV-2a "Asian-like markers" (LC214970)	99.09	99.49	99.49	99.32	99.60	99.32	99.26	99.32	99.37	99.37	99.37	99.37	99.89	99.89	99.83	ID	99.83	99.83	99.32	98.97	98.97	98.97	98.97	98.97	98.97	98.97	98.97	98.97	98.97	98.97	98.97	98.97	98.97	98.97	98.80	98.80	98.80	99.32	99.32	99.32	99.32	99.32	99.32	99.32	99.32	99.32	99.14	99.32	99.14	99.14	99.32	99.32	99.32	99.32
**CPV-2a "Asian-like markers" (PX146839)**	99.20	99.60	99.60	99.43	99.60	99.43	99.37	99.43	99.49	99.49	99.49	99.49	99.77	99.77	99.72	99.77	ID	100.00	99.49	99.14	99.14	99.14	99.14	99.14	99.14	99.14	99.14	99.14	99.14	99.14	99.14	99.14	99.14	99.14	98.97	98.97	98.97	99.49	99.49	99.49	99.49	99.49	99.49	99.49	99.49	99.49	99.32	99.49	99.32	99.32	99.49	99.49	99.49	99.49
**CPV-2a "Asian-like markers" (PX146844)**	99.20	99.60	99.60	99.43	99.60	99.43	99.37	99.43	99.49	99.49	99.49	99.49	99.77	99.77	99.72	99.77	100.00	ID	99.49	99.14	99.14	99.14	99.14	99.14	99.14	99.14	99.14	99.14	99.14	99.14	99.14	99.14	99.14	99.14	98.97	98.97	98.97	99.49	99.49	99.49	99.49	99.49	99.49	99.49	99.49	99.49	99.32	99.49	99.32	99.32	99.49	99.49	99.49	99.49
CPV-2b (KR559895)	99.26	99.26	99.26	99.26	99.43	99.15	99.09	99.15	99.20	99.20	99.20	99.20	99.03	99.03	98.97	99.03	99.15	99.15	ID	99.66	99.66	99.66	99.66	99.66	99.66	99.66	99.66	99.66	99.66	99.66	99.66	99.66	99.66	99.66	99.49	99.49	99.49	99.32	99.32	99.32	99.32	99.32	99.32	99.32	99.32	99.32	99.66	99.83	99.66	98.97	99.14	99.14	99.14	99.14
CPV-2b (MT353761)	99.43	99.43	99.43	99.43	99.60	99.32	99.26	99.32	99.37	99.37	99.37	99.37	99.20	99.20	99.15	99.20	99.32	99.32	99.49	ID	100.00	100.00	100.00	100.00	100.00	100.00	100.00	100.00	100.00	100.00	100.00	100.00	100.00	100.00	99.83	99.83	99.83	98.97	98.97	98.97	98.97	98.97	98.97	98.97	98.97	98.97	99.32	99.49	99.32	98.63	98.80	98.80	98.80	98.80
CPV-2b (MT353762)	99.26	99.37	99.37	99.26	99.43	99.26	99.20	99.26	99.32	99.32	99.32	99.32	99.03	99.03	98.97	99.03	99.26	99.26	99.32	99.72	ID	100.00	100.00	100.00	100.00	100.00	100.00	100.00	100.00	100.00	100.00	100.00	100.00	100.00	99.83	99.83	99.83	98.97	98.97	98.97	98.97	98.97	98.97	98.97	98.97	98.97	99.32	99.49	99.32	98.63	98.80	98.80	98.80	98.80
CPV-2b (MT353763)	99.37	99.49	99.49	99.37	99.54	99.37	99.32	99.37	99.43	99.43	99.43	99.43	99.15	99.15	99.09	99.15	99.26	99.26	99.43	99.83	99.77	ID	100.00	100.00	100.00	100.00	100.00	100.00	100.00	100.00	100.00	100.00	100.00	100.00	99.83	99.83	99.83	98.97	98.97	98.97	98.97	98.97	98.97	98.97	98.97	98.97	99.32	99.49	99.32	98.63	98.80	98.80	98.80	98.80
**CPV-2b (PX146846)**	99.37	99.37	99.37	99.37	99.54	99.26	99.20	99.26	99.32	99.32	99.32	99.32	99.15	99.15	99.09	99.15	99.37	99.37	99.43	99.94	99.77	99.77	ID	100.00	100.00	100.00	100.00	100.00	100.00	100.00	100.00	100.00	100.00	100.00	99.83	99.83	99.83	98.97	98.97	98.97	98.97	98.97	98.97	98.97	98.97	98.97	99.32	99.49	99.32	98.63	98.80	98.80	98.80	98.80
**CPV-2b (PX146847)**	99.37	99.37	99.37	99.37	99.54	99.26	99.20	99.26	99.32	99.32	99.32	99.32	99.15	99.15	99.09	99.15	99.26	99.26	99.43	99.94	99.66	99.77	99.89	ID	100.00	100.00	100.00	100.00	100.00	100.00	100.00	100.00	100.00	100.00	99.83	99.83	99.83	98.97	98.97	98.97	98.97	98.97	98.97	98.97	98.97	98.97	99.32	99.49	99.32	98.63	98.80	98.80	98.80	98.80
**CPV-2b (PX146848)**	99.37	99.37	99.37	99.37	99.54	99.26	99.20	99.26	99.32	99.32	99.32	99.32	99.15	99.15	99.09	99.15	99.37	99.37	99.43	99.94	99.77	99.77	100.00	99.89	ID	100.00	100.00	100.00	100.00	100.00	100.00	100.00	100.00	100.00	99.83	99.83	99.83	98.97	98.97	98.97	98.97	98.97	98.97	98.97	98.97	98.97	99.32	99.49	99.32	98.63	98.80	98.80	98.80	98.80
**CPV-2b (PX146849)**	99.37	99.37	99.37	99.37	99.54	99.26	99.20	99.26	99.32	99.32	99.32	99.32	99.15	99.15	99.09	99.15	99.26	99.26	99.43	99.94	99.66	99.77	99.89	100.00	99.89	ID	100.00	100.00	100.00	100.00	100.00	100.00	100.00	100.00	99.83	99.83	99.83	98.97	98.97	98.97	98.97	98.97	98.97	98.97	98.97	98.97	99.32	99.49	99.32	98.63	98.80	98.80	98.80	98.80
**CPV-2b (PX146850)**	99.43	99.43	99.43	99.43	99.60	99.32	99.26	99.32	99.37	99.37	99.37	99.37	99.20	99.20	99.15	99.20	99.32	99.32	99.49	100.00	99.72	99.83	99.94	99.94	99.94	99.94	ID	100.00	100.00	100.00	100.00	100.00	100.00	100.00	99.83	99.83	99.83	98.97	98.97	98.97	98.97	98.97	98.97	98.97	98.97	98.97	99.32	99.49	99.32	98.63	98.80	98.80	98.80	98.80
**CPV-2b (PX146851)**	99.37	99.37	99.37	99.37	99.54	99.26	99.20	99.26	99.32	99.32	99.32	99.32	99.15	99.15	99.09	99.15	99.26	99.26	99.43	99.94	99.66	99.77	99.89	100.00	99.89	100.00	99.94	ID	100.00	100.00	100.00	100.00	100.00	100.00	99.83	99.83	99.83	98.97	98.97	98.97	98.97	98.97	98.97	98.97	98.97	98.97	99.32	99.49	99.32	98.63	98.80	98.80	98.80	98.80
**CPV-2b (PX146852)**	99.43	99.43	99.43	99.43	99.60	99.32	99.26	99.32	99.37	99.37	99.37	99.37	99.20	99.20	99.15	99.20	99.32	99.32	99.49	100.00	99.72	99.83	99.94	99.94	99.94	99.94	100.00	99.94	ID	100.00	100.00	100.00	100.00	100.00	99.83	99.83	99.83	98.97	98.97	98.97	98.97	98.97	98.97	98.97	98.97	98.97	99.32	99.49	99.32	98.63	98.80	98.80	98.80	98.80
**CPV-2b (PX146853)**	99.43	99.43	99.43	99.43	99.60	99.32	99.26	99.32	99.37	99.37	99.37	99.37	99.20	99.20	99.15	99.20	99.32	99.32	99.49	100.00	99.72	99.83	99.94	99.94	99.94	99.94	100.00	99.94	100.00	ID	100.00	100.00	100.00	100.00	99.83	99.83	99.83	98.97	98.97	98.97	98.97	98.97	98.97	98.97	98.97	98.97	99.32	99.49	99.32	98.63	98.80	98.80	98.80	98.80
**CPV-2b (PX146854)**	99.37	99.37	99.37	99.37	99.54	99.26	99.20	99.26	99.32	99.32	99.32	99.32	99.15	99.15	99.09	99.15	99.26	99.26	99.43	99.94	99.66	99.77	99.89	100.00	99.89	100.00	99.94	100.00	99.94	99.94	ID	100.00	100.00	100.00	99.83	99.83	99.83	98.97	98.97	98.97	98.97	98.97	98.97	98.97	98.97	98.97	99.32	99.49	99.32	98.63	98.80	98.80	98.80	98.80
**CPV-2b (PX146855)**	99.37	99.37	99.37	99.37	99.54	99.26	99.20	99.26	99.32	99.32	99.32	99.32	99.15	99.15	99.09	99.15	99.26	99.26	99.43	99.94	99.66	99.77	99.89	100.00	99.89	100.00	99.94	100.00	99.94	99.94	100.00	ID	100.00	100.00	99.83	99.83	99.83	98.97	98.97	98.97	98.97	98.97	98.97	98.97	98.97	98.97	99.32	99.49	99.32	98.63	98.80	98.80	98.80	98.80
**CPV-2b (PX146856)**	99.37	99.37	99.37	99.37	99.54	99.26	99.20	99.26	99.32	99.32	99.32	99.32	99.15	99.15	99.09	99.15	99.26	99.26	99.43	99.94	99.66	99.77	99.89	100.00	99.89	100.00	99.94	100.00	99.94	99.94	100.00	100.00	ID	100.00	99.83	99.83	99.83	98.97	98.97	98.97	98.97	98.97	98.97	98.97	98.97	98.97	99.32	99.49	99.32	98.63	98.80	98.80	98.80	98.80
**CPV-2b (PX146857)**	99.37	99.37	99.37	99.37	99.54	99.26	99.20	99.26	99.32	99.32	99.32	99.32	99.15	99.15	99.09	99.15	99.26	99.26	99.43	99.94	99.66	99.77	99.89	100.00	99.89	100.00	99.94	100.00	99.94	99.94	100.00	100.00	100.00	ID	99.83	99.83	99.83	98.97	98.97	98.97	98.97	98.97	98.97	98.97	98.97	98.97	99.32	99.49	99.32	98.63	98.80	98.80	98.80	98.80
**CPV-2b (PX146858)**	99.20	99.20	99.20	99.20	99.37	99.09	99.15	99.09	99.15	99.15	99.15	99.15	98.97	98.97	98.92	98.97	99.09	99.09	99.26	99.77	99.49	99.60	99.72	99.72	99.72	99.72	99.77	99.72	99.77	99.77	99.72	99.72	99.72	99.72	ID	100.00	100.00	98.80	98.80	98.80	98.80	98.80	98.80	98.80	98.80	98.80	99.14	99.32	99.14	98.46	98.63	98.63	98.63	98.63
**CPV-2b (PX146859)**	99.20	99.20	99.20	99.20	99.37	99.09	99.15	99.09	99.15	99.15	99.15	99.15	98.97	98.97	98.92	98.97	99.09	99.09	99.26	99.77	99.49	99.60	99.72	99.72	99.72	99.72	99.77	99.72	99.77	99.77	99.72	99.72	99.72	99.72	100.00	ID	100.00	98.80	98.80	98.80	98.80	98.80	98.80	98.80	98.80	98.80	99.14	99.32	99.14	98.46	98.63	98.63	98.63	98.63
**CPV-2b (PX146860)**	99.20	99.20	99.20	99.20	99.37	99.09	99.15	99.09	99.15	99.15	99.15	99.15	98.97	98.97	98.92	98.97	99.09	99.09	99.26	99.77	99.49	99.60	99.72	99.72	99.72	99.72	99.77	99.72	99.77	99.77	99.72	99.72	99.72	99.72	100.00	100.00	ID	98.80	98.80	98.80	98.80	98.80	98.80	98.80	98.80	98.80	99.14	99.32	99.14	98.46	98.63	98.63	98.63	98.63
"Asian-like" CPV-2b (ON733252)	98.86	99.03	99.03	98.86	99.03	98.97	98.80	98.97	98.92	98.92	98.92	98.92	99.09	99.09	99.03	99.09	99.32	99.32	98.92	99.09	99.03	99.03	99.15	99.15	99.15	99.15	99.09	99.15	99.09	99.09	99.15	99.15	99.15	99.15	99.09	99.09	99.09	ID	100.00	100.00	100.00	100.00	100.00	100.00	100.00	100.00	98.97	99.14	98.97	99.66	99.83	99.83	99.83	99.83
"Asian-like" CPV-2b (OR825360)	98.92	99.09	99.09	98.92	99.09	98.80	98.75	98.80	98.86	98.86	98.86	98.86	99.15	99.15	99.09	99.15	99.37	99.37	98.97	99.15	99.09	99.09	99.20	99.20	99.20	99.20	99.15	99.20	99.15	99.15	99.20	99.20	99.20	99.20	99.15	99.15	99.15	99.83	ID	100.00	100.00	100.00	100.00	100.00	100.00	100.00	98.97	99.14	98.97	99.66	99.83	99.83	99.83	99.83
"Asinal-like" CPV-2b (ON677437)	98.80	98.97	98.97	98.80	98.97	98.92	98.75	98.92	98.86	98.86	98.86	98.86	99.03	99.03	98.97	99.03	99.26	99.26	98.86	99.03	98.97	98.97	99.09	99.09	99.09	99.09	99.03	99.09	99.03	99.03	99.09	99.09	99.09	99.09	99.03	99.03	99.03	99.94	99.77	ID	100.00	100.00	100.00	100.00	100.00	100.00	98.97	99.14	98.97	99.66	99.83	99.83	99.83	99.83
"Asian-like" CPV-2b (OR463607)	98.80	98.97	98.97	98.80	98.97	98.92	98.75	98.92	98.86	98.86	98.86	98.86	99.03	99.03	98.97	99.03	99.26	99.26	98.86	99.03	98.97	98.97	99.09	99.09	99.09	99.09	99.03	99.09	99.03	99.03	99.09	99.09	99.09	99.09	99.03	99.03	99.03	99.94	99.77	100.00	ID	100.00	100.00	100.00	100.00	100.00	98.97	99.14	98.97	99.66	99.83	99.83	99.83	99.83
**"Asian-like" CPV-2b (PX146861)**	98.86	99.03	99.03	98.86	99.03	98.97	98.80	98.97	98.92	98.92	98.92	98.92	99.09	99.09	99.03	99.09	99.32	99.32	98.92	99.09	99.03	99.03	99.15	99.15	99.15	99.15	99.09	99.15	99.09	99.09	99.15	99.15	99.15	99.15	99.09	99.09	99.09	100.00	99.83	99.94	99.94	ID	100.00	100.00	100.00	100.00	98.97	99.14	98.97	99.66	99.83	99.83	99.83	99.83
**"Asian-like" CPV-2b (PX146862)**	98.86	99.03	99.03	98.86	99.03	98.97	98.80	98.97	98.92	98.92	98.92	98.92	99.09	99.09	99.03	99.09	99.32	99.32	98.92	99.09	99.03	99.03	99.15	99.15	99.15	99.15	99.09	99.15	99.09	99.09	99.15	99.15	99.15	99.15	99.09	99.09	99.09	100.00	99.83	99.94	99.94	100.00	ID	100.00	100.00	100.00	98.97	99.14	98.97	99.66	99.83	99.83	99.83	99.83
**"Asian-like" CPV-2b (PX146863)**	98.86	99.03	99.03	98.86	99.03	98.97	98.80	98.97	98.92	98.92	98.92	98.92	99.09	99.09	99.03	99.09	99.32	99.32	98.92	99.09	99.03	99.03	99.15	99.15	99.15	99.15	99.09	99.15	99.09	99.09	99.15	99.15	99.15	99.15	99.09	99.09	99.09	100.00	99.83	99.94	99.94	100.00	100.00	ID	100.00	100.00	98.97	99.14	98.97	99.66	99.83	99.83	99.83	99.83
**"Asian-like" CPV-2b (PX146864)**	98.86	99.03	99.03	98.86	99.03	98.97	98.80	98.97	98.92	98.92	98.92	98.92	99.09	99.09	99.03	99.09	99.32	99.32	98.92	99.09	99.03	99.03	99.15	99.15	99.15	99.15	99.09	99.15	99.09	99.09	99.15	99.15	99.15	99.15	99.09	99.09	99.09	100.00	99.83	99.94	99.94	100.00	100.00	100.00	ID	100.00	98.97	99.14	98.97	99.66	99.83	99.83	99.83	99.83
**"Asian-like" CPV-2b (PX146865)**	98.92	99.09	99.09	98.92	99.09	98.92	98.86	98.92	98.97	98.97	98.97	98.97	99.15	99.15	99.09	99.15	99.37	99.37	98.97	99.15	99.09	99.09	99.20	99.20	99.20	99.20	99.15	99.20	99.15	99.15	99.20	99.20	99.20	99.20	99.15	99.15	99.15	99.94	99.89	99.89	99.89	99.94	99.94	99.94	99.94	ID	98.97	99.14	98.97	99.66	99.83	99.83	99.83	99.83
CPV-2c (FJ005195)	99.43	99.43	99.43	99.43	99.60	99.32	99.26	99.32	99.37	99.37	99.37	99.37	99.20	99.20	99.15	99.20	99.32	99.32	99.60	99.66	99.49	99.60	99.60	99.60	99.60	99.60	99.66	99.60	99.66	99.66	99.60	99.60	99.60	99.60	99.43	99.43	99.43	99.09	99.15	99.03	99.03	99.09	99.09	99.09	99.09	99.15	ID	99.83	99.66	98.97	99.14	99.14	99.14	99.14
CPV-2c (KP682511)	99.37	99.37	99.37	99.37	99.54	99.26	99.20	99.26	99.32	99.32	99.32	99.32	99.15	99.15	99.09	99.15	99.26	99.26	99.54	99.60	99.43	99.54	99.54	99.54	99.54	99.54	99.60	99.54	99.60	99.60	99.54	99.54	99.54	99.54	99.37	99.37	99.37	99.03	99.09	98.97	98.97	99.03	99.03	99.03	99.03	99.09	99.83	ID	99.83	99.14	99.32	99.32	99.32	99.32
CPV-2c (KP682530)	99.37	99.37	99.37	99.37	99.54	99.26	99.20	99.26	99.32	99.32	99.32	99.32	99.15	99.15	99.09	99.15	99.26	99.26	99.54	99.60	99.43	99.54	99.54	99.54	99.54	99.54	99.60	99.54	99.60	99.60	99.54	99.54	99.54	99.54	99.37	99.37	99.37	99.03	99.09	98.97	98.97	99.03	99.03	99.03	99.03	99.09	99.83	99.77	ID	98.97	99.14	99.14	99.14	99.14
"Asian" CPV-2c (OP595742)	98.86	99.03	99.03	98.86	99.03	98.86	98.80	98.86	98.92	98.92	98.92	98.92	99.09	99.09	99.03	99.09	99.32	99.32	98.92	99.09	99.03	99.03	99.15	99.15	99.15	99.15	99.09	99.15	99.09	99.09	99.15	99.15	99.15	99.15	98.86	98.86	98.86	99.54	99.49	99.49	99.49	99.54	99.54	99.54	99.54	99.60	99.20	99.15	99.15	ID	99.83	99.83	99.83	99.83
"Asian" CPV-2c (MN832850)	98.80	98.97	98.97	98.80	98.97	98.80	98.75	98.80	98.86	98.86	98.86	98.86	99.03	99.03	98.97	99.03	99.26	99.26	98.86	99.03	98.97	98.97	99.09	99.09	99.09	99.09	99.03	99.09	99.03	99.03	99.09	99.09	99.09	99.09	98.80	98.80	98.80	99.49	99.43	99.43	99.43	99.49	99.49	99.49	99.49	99.54	99.15	99.09	99.09	99.83	ID	100.00	100.00	100.00
"Asian" CPV-2c (MW659469)	98.92	99.09	99.09	98.92	99.09	98.92	98.86	98.92	98.97	98.97	98.97	98.97	99.15	99.15	99.09	99.15	99.37	99.37	98.97	99.15	99.09	99.09	99.20	99.20	99.20	99.20	99.15	99.20	99.15	99.15	99.20	99.20	99.20	99.20	98.92	98.92	98.92	99.60	99.54	99.54	99.54	99.60	99.60	99.60	99.60	99.66	99.26	99.20	99.20	99.94	99.89	ID	100.00	100.00
"Asian" CPV-2c (OR463608)	98.75	98.92	98.92	98.75	98.92	98.86	98.69	98.86	98.80	98.80	98.80	98.80	98.97	98.97	98.92	98.97	99.20	99.20	98.80	98.97	98.92	98.92	99.03	99.03	99.03	99.03	98.97	99.03	98.97	98.97	99.03	99.03	99.03	99.03	98.86	98.86	98.86	99.77	99.60	99.83	99.83	99.77	99.77	99.77	99.77	99.72	99.09	99.03	99.03	99.66	99.60	99.72	ID	100.00
**"Asian" CPV-2c (PX146866)**	98.86	99.03	99.03	98.86	99.03	98.86	98.80	98.86	98.92	98.92	98.92	98.92	99.09	99.09	99.03	99.09	99.32	99.32	98.92	99.09	99.03	99.03	99.15	99.15	99.15	99.15	99.09	99.15	99.09	99.09	99.15	99.15	99.15	99.15	98.97	98.97	98.97	99.66	99.60	99.60	99.60	99.66	99.66	99.66	99.66	99.72	99.20	99.15	99.15	99.89	99.83	99.94	100.00	ID

Sequence matrix identity (%)

98 98.2 98.4 98.6 98.8 99 99.2 99.4 99.6 99.8 100

98 98.2 98.4 98.6 98.8 99 99.2 99.4 99.6 99.8 100

Figure S4. Similarity identity matrix of the *VP2* nucleotide sequences (highlighted in green/yellow, lower triangle) and amino acid sequences (highlighted in red/blue, upper triangle) of the CPV-2 isolates from this study and reference strains. Slovak CPV-2a isolates showed 99.43 – 100% nucleotide and 99.32 – 100% amino acid identity with CPV-2a reference strains, while identities to Asian-like CPV-2a strains were slightly lower (99.20% – 99.49% nucleotide identity; 99.14% – 99.49% amino acid identity). CPV-2a isolates with "Asian-like markers" from this study showed 99.72% – 99.77% nucleotide and 99.66% – 99.83% amino acid identity with CPV-2a "Asian-like markers" reference strains. CPV-2b isolates exhibited 99.26 % – 100% nucleotide and 99.49% – 100% amino acid identity with CPV-2b reference strains, and 99.03% – 99.20% nucleotide and 98.80% – 98.97% amino acid identity to "Asian-like" CPV-2b strains. "Asian-like" CPV-2b from Slovakia demonstrated 99.83% – 100% nucleotide and complete amino acid identity (100%) to the corresponding reference "Asian-like" CPV-2b strains. The single "Asian" CPV-2c sequence obtained in this study demonstrated 99.3% – 99.83% nucleotide and 99.14% – 99.32% amino acid identity with CPV-2c reference strains, while identity with "Asian" CPV-2c reference strains reached 99.83% –100%. Sequences identified in this study are displayed in bold.

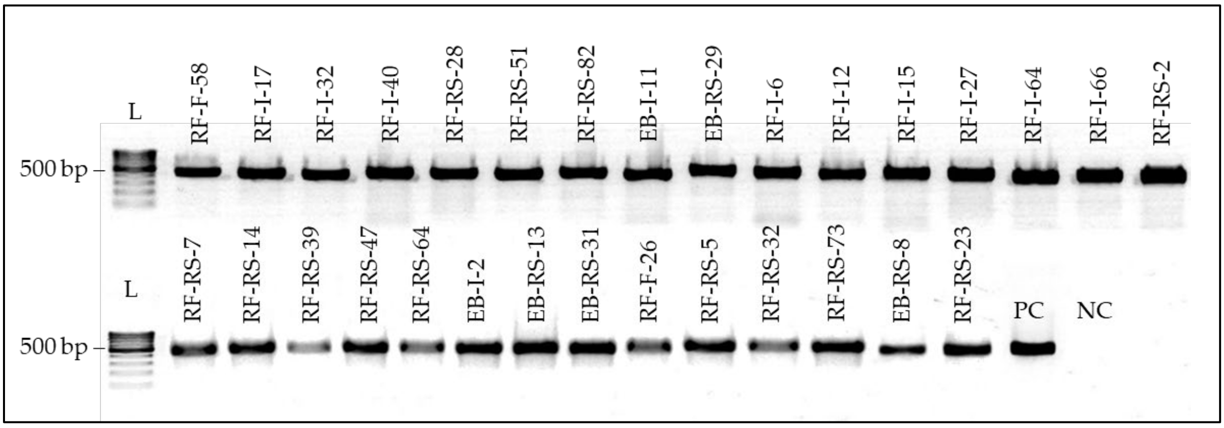

Figure S5. Confirmation of CPV isolation on MDCK cell line. Gel electrophoresis of PCR products targeting the *VP2* gene (573 bp) from MDCK cell culture supernatants. Viral DNA was detected after the third passage, confirming successful CPV isolation and replication. L – 100 bp DNA ladder; PC – positive control (CPV-positive dog; OR825360); NC – negative control (non-infected MDCK).

Supplementary Tables

Table S1. List of reference sequences used in this study for phylogenetic analysis (accessed August 10, 2025).

GenBank accession number	CPV-2 variant	Strain / Isolate	Region	Collection year
EU659116	CPV-2	CPV-5.us.79	USA	1979
M23255	CPV-2	CPV-d Cornell #320	USA	1988
DQ354068	CPV-2a	RPPV	China	2004
JN867615	CPV-2a	CPV/Raccoon/GA/287/08	USA	2009
JQ686671	CPV-2a	CPV-2a	China	2011
JX660690	CPV-2a	SC02/2011	China	2011
KF366250	CPV-2a	CPV/915-H	India	2013
KJ813871	CPV-2a	CPV/Raccoon/VT/460/2013	USA	2013
KR002792	CPV-2a	CPV/CN/SH1/2013	China	2013
KR002802	CPV-2a	CPV/CN/SD9/2014	China	2014
KX434454	CPV-2a	CPV_IZSSI_29451_09	Italy	2009
LC214970	CPV-2a	CPV/dog/HCM/22/2013	Vietnam	2013
LC621934	CPV-2a	CPV2-181	Japan	2016
MG434741	CPV-2a	CPV_IZSSI_PA5610/2017	Italy	2017
MG434742	CPV-2a	CPV_IZSSI_PA10388/2017	Italy	2017
MG763189	CPV-2a	CPV-L	China	2014
MH213135	CPV-2a	17Ra5	South Korea	2018
MH213140	CPV-2a	17Ra171	South Korea	2018
MK413740	CPV-2a	CPV-2a_PA30636/17	Italy	2017
MK413741	CPV-2a	CPV-2a_PA31209/17	Italy	2017
MK675667	CPV-2a	CPV2a/18SY0902	China	2018
MK895485	CPV-2a	IZSSI_PA1464/19_idUV6	Nigeria	2018
MT981022	CPV-2a	IZSSI_PA17245/18	Italy	2018
ON185547	CPV-2a	CPV_061_HU VP2	Hungary	2020
OP208806	CPV-2a	CPV-2a/pangolin/China/P2/20	China	2020
OP985523	CPV-2a	CPV2/NGR-1	Nigeria	2014
JX411926	CPV-2b	CPV/Stone marten/PT10	Portugal	2010
KJ813863	CPV-2b	CPV/Puma/ND/M96/2013	USA	2013
KP682512	CPV-2b	158	Spain	2012
KR559895	CPV-2b	PT077/13	Portugal	2013
MH106699	CPV-2b	CPV-BJL2	China	2015
MH476588	CPV-2b	Canine/China/19/2017	China	2017
MH491898	CPV-2b	31179_Italy_Lazio_11_09_2015	Italy	2015
MH614271	CPV-2b	Majella/pack7/07	Italy	2017
MK348090	CPV-2b	CPV-2b/392/2013	Italy	2013
MK348102	CPV-2b	CPV-2b/1418/2016	Italy	2016
MK413742	CPV-2b	CPV-2b_PA13600/17	Italy	2017
MT353761	CPV-2b	CPV2b/ITALY/2019/Wolf/415/19-3530	Italy	2019
MT353762	CPV-2b	CPV2b/ITALY/2019/Badger//289/19-5860	Italy	2019
MT353763	CPV-2b	CPV2b/ITALY/2019/Bmarten/289/19-5771	Italy	2019
MT353764	CPV-2b	CPV2b/ITALY/2019/Badger/289/19-5624	Italy	2019

MT648206	CPV-2b	CPV-AHmas3	China	2018
MT952857	CPV-2b	TR/HSS20	Turkey	2016
OM721655	CPV-2b	CPV-2b-K5-TR	Turkey	2019
ON677437	CPV-2b	CPV-2b_IZSSI_2022PA2773	Italy	2022
ON733252	CPV-2b	FR1/CPV2-2021-HUN	Hungary	2021
OR463607	CPV-2b	IZSSI_2022PA15678idMeF	Italy	2022
OR825360	CPV-2b	Slovakia/Kosice/47/2022	Slovakia	2022
KJ813848	CPV-2c	CPV/Bobcat/ND/1162/2013	USA	2013
KJ813854	CPV-2c	CPV/Puma/ND/F205/2013	USA	2013
KM457120	CPV-2c	UY242	Uruguay	2010
KP682511	CPV-2c	150	Spain	2011
KP682521	CPV-2c	417	Spain	2009
KP682527	CPV-2c	172	Spain	2011
KP682530	CPV-2c	160	Spain	2012
KU508691	CPV-2c	HB	Australia	2015
KU662351	CPV-2c	Sm14/PT/08	Portugal	2008
KY073269	CPV-2c	UFMT	Brazil	2015
MF177227	CPV-2c	202-09	France	2009
MF510157	CPV-2c	CPV_IZSSI_2743_17	Italy	2017
MK144544	CPV-2c	K01708-1	South Korea	2017
MN832850	CPV-2c	Taiwan/2018	Taiwan	2018
MT353760	CPV-2c	CPV2c/ITALY/2019/Badger/289/19-5518	Italy	2019
MT585709	CPV-2c	13P.CL	Chile	2019
MT648203	CPV-2c	CPV-AHhf27	China	2019
MT840294	CPV-2c	IZSSI_PA1464/19_idYV7_TR_4A72	Nigeria	2018
MW589468	CPV-2c	TRC-B90/TH/2020	Thailand	2020
MW659469	CPV-2c	157/2019	Romania	2019
OK094443	CPV-2	VNUA/CPV380-Hanoi	Vietnam	2020
OM100701	CPV-2c	EGY-FVMVL-36/2019	Egypt	2020
OM640098	CPV-2c	FM4	Canada	2018
OM937842	CPV-2c	16d	Ethiopia	2021
ON322838	CPV-2c	HN-100	China	2021
OP588002	CPV-2c	IZSVe_21/31130-1_dog_ITA	Italy	2021
OP595742	CPV-2c	IZSVe_22/14754-6_gray_wolf_ITA	Italy	2022
OP611196	CPV-2c	Gab-9	Gabon	2019
OQ092740	CPV-2c	CPV/INDIA/AP45	India	2022
OR399577	CPV-2c	CPV2/2022/3	China	2022/2023
KX434462	FPV	FPV_IZSSI_42807_15	Italy	2015

Table S2. List of sequences producing significant alignments with the partial *COI* gene sequence from fecal samples using the NCBI BLASTn algorithm (accessed March 15, 2025). A fine line indicates the 98% cut-off for species confirmation.

GenBank accession number	Name	Pairwise identity [%]	Query coverage [%]
NC_008434.1	*Vulpes vulpes*	100.00	100
JN711443.1	*Vulpes vulpes*	99.60	100
GQ374180.1	*Vulpes vulpes*	99.60	100
MN122913.1	*Vulpes vulpes*	99.60	100
KT448287.1	*Vulpes vulpes*	99.60	100
KF387633.1	*Vulpes vulpes*	99.40	100
OZ067330.1	*Vulpes vulpes*	99.01	100
KM657026.1	*Vulpes vulpes*	98.41	100
KM657025.1	*Vulpes vulpes*	98.41	100
KP342452.1	*Vulpes vulpes*	98.21	100
KM657024.1	*Vulpes vulpes*	98.21	100
KM657027.1	*Vulpes vulpes*	98.21	100
KM657023.1	*Vulpes vulpes*	98.01	100
KM657029.1	*Vulpes vulpes*	98.01	100
KM657028.1	*Vulpes vulpes*	98.01	100
NC_087722.1	*Vulpes rueppelii*	97.81	82
AY894421.1	*Vulpes lagopus*	92.72	82
LT559678.1	*Vulpes lagopus*	92.64	100
LT559978.1	*Vulpes lagopus*	92.45	100
LT559882.1	*Vulpes lagopus*	92.45	100
LT559498.1	*Vulpes lagopus*	92.45	100
LT559750.1	*Vulpes lagopus*	92.45	100
LT560026.1	*Vulpes lagopus*	92.45	100
LT560050.1	*Vulpes lagopus*	92.45	100
LT559798.1	*Vulpes lagopus*	92.45	100
NC_026529.1	*Vulpes lagopus*	92.45	100
LT559510.1	*Vulpes lagopus*	92.45	100
LT559990.1	*Vulpes lagopus*	92.45	100
LT560014.1	*Vulpes lagopus*	92.45	100
LT559546.1	*Vulpes lagopus*	92.45	100
MT883492.1	*Vulpes lagopus*	92.45	100
LT559714.1	*Vulpes lagopus*	92.45	100
OR880604.1	*Vulpes lagopus*	92.45	100
LT559606.1	*Vulpes lagopus*	92.45	100
LT559654.1	*Vulpes lagopus*	92.45	100
LT559822.1	*Vulpes lagopus*	92.45	100
LT559954.1	*Vulpes lagopus*	92.45	100
LT559858.1	*Vulpes lagopus*	92.45	100
LT559582.1	*Vulpes lagopus*	92.45	100
LT560062.1	*Vulpes lagopus*	92.45	100
LT559762.1	*Vulpes lagopus*	92.45	100
LT559738.1	*Vulpes lagopus*	92.45	100
LT559906.1	*Vulpes lagopus*	92.45	100

LT560002.1	*Vulpes lagopus*	92.45	100
KP342451.1	*Vulpes lagopus*	92.45	100
LT559534.1	*Vulpes lagopus*	92.45	100
LT559834.1	*Vulpes lagopus*	92.45	100
LT559630.1	*Vulpes lagopus*	92.45	100
LT559930.1	*Vulpes lagopus*	92.45	100
LT559966.1	*Vulpes lagopus*	92.45	100
AH014073.2	*Vulpes lagopus*	92.45	100
LT559594.1	*Vulpes lagopus*	92.45	100
PV090785.1	*Vulpes velox*	92.45	100
KT448286.1	*Vulpes lagopus*	92.45	100
LT559570.1	*Vulpes lagopus*	92.45	100
NC_023958.1	*Vulpes corsac*	92.45	100
LT560038.1	*Vulpes lagopus*	92.45	100
LT559846.1	*Vulpes lagopus*	92.45	100
LT559487.1	*Vulpes lagopus*	92.45	100
LT559894.1	*Vulpes lagopus*	92.45	100
LT559870.1	*Vulpes lagopus*	92.45	100
LT559642.1	*Vulpes lagopus*	92.45	100
OR880606.1	*Vulpes lagopus*	92.45	100
LT559702.1	*Vulpes lagopus*	92.45	100
LT559690.1	*Vulpes lagopus*	92.25	100
LT559522.1	*Vulpes lagopus*	92.25	100
LT559726.1	*Vulpes lagopus*	92.25	100
LT559942.1	*Vulpes lagopus*	92.25	100
LT559666.1	*Vulpes lagopus*	92.25	100
LT559810.1	*Vulpes lagopus*	92.25	100
LT559558.1	*Vulpes lagopus*	92.25	100
LT559618.1	*Vulpes lagopus*	92.25	100
LT559918.1	*Vulpes lagopus*	92.25	100
OR880609.1	*Vulpes lagopus*	92.25	100
LT559774.1	*Vulpes lagopus*	92.25	100
LT559786.1	*Vulpes lagopus*	92.25	100
AF028205.1	*Vulpes macrotis*	91.99	82
AY894422.1	*Vulpes corsac*	91.26	82
NC_027935.1	*Vulpes ferrilata*	91.25	100
OR880608.1	*Vulpes lagopus*	89.28	69
PV090783.1	*Vulpes cana*	89.26	100
KJ603240.1	*Vulpes zerda*	88.84	100
NC_070063.1	*Vulpes chama*	88.45	100
ON756054.3	*Vulpes chama*	88.45	100

Table S3. Statistical analysis of CPV DNA copy numbers normalized to input material by host and sample type.

A) CPV-2 DNA copy numbers by sample type (pooled species)

Host species	Sample type	Pos/Tested (prevalence; 95%CI[1])	Median (Q1 – Q3)	Viral load range (min – max)
**foxes + badgers**	intestine	11/86 (12.8%; 7.3 – 21.5)	9.75×10^3 (7.15×10^3 – 6.06×10^4)	2.08×10^3 – 5.73×10^5
	rectal swab	17/123 (13.8%; 8.8 – 21)	4.54×10^2 (2.87×10^2 – 6.7×10^3)	8.7×10^1 – 6.7×10^3
**foxes**	feces	2/65 (3.1%; 0.5 – 10.5)	4.27×10^2 (2.23×10^2 – 6.31×10^2)	2.23×10^2 – 6.31×10^2

B) Pairwise comparisons of CPV DNA copy numbers[2]

Host species	Sample type	Pos/Tested (prevalence; 95%CI[1])	Median (Q1 – Q3)	Viral load range (min – max)	Test (p value)	Comment
fox vs badger	rectal swab vs rectal swab	13/84 (15.5%; 9.3 – 24.7)<br>4/39 (10.3%; 4.1 – 23.6)	4.54×10^2 (2.89×10^2 – 2.17×10^3)<br>4.75×10^2 (1.31×10^2 – 2.02×10^3)	2.02×10^2 – 6.7×10^3<br>8.7×10^1 – 2.47×10^3	Mann–Whitney U ($p = 0.6235$)	no significant
fox vs badger	intestine vs intestine	9/72 (12.5%; 6.7 – 22.1)<br>2/14 (14.3%; 2.5 – 39.9)	8.74×10^3 (5.015×10^3 – 2.67×10^4)<br>3.67×10^5 (1.61×10^5 – 5.73×10^5)	2.08×10^3 – 6.06×10^4<br>1.61×10^5 – 5.73×10^5	Mann–Whitney U ($p = 0.0364$)	badger > fox

[1] The Wilson/Brown method (95% confidence interval, CI) was carried out on a total of positive samples.

[2] Fecal samples, available only from red foxes, were excluded from pairwise statistical analysis.

Please note that DNA copy numbers were normalized per input material (copies/mg for intestine and fecal samples or copies/μL for rectal swabs). Statistical significance was set at $p < 0.05$.

Table S4. Amino acid and nucleotide profile observed in CPV-2 sequences from wild carnivores identified in this study. Non-synonymous mutations are displayed in bold.

**Host animal**	**Sample ID**	**Isolate**	**GenBank acc. number**	**CPV-2 variant**	**Phylogeny cluster**	**VP2 aa residues (Nt position)**								
						**5 (13-15)**	**267 (799-801)**	**297 (889-891)**	**324 (970-972)**	**370 (1108-1110)**	**426 (1276-1278)**	**440 (1318-1320)**	**447 (1339-1341)**	**552 (1654-1656)**
red fox	RF-F-58	CPV-2a/Slovakia:Zilina Region/2025/red fox/RF-F-58	PX146837	CPV-2a	CPV-2a	A (GCA)	F (TTT)	A (GCT)	Y (TAT)	Q (CAA)	N (AAT)	T (ACA)	I (ATA)	S (AGT)
red fox	RF-I-17	CVP-2a/Slovakia:Presov Region/2023/red fox/RF-I-17	PX146838	CPV-2a	CPV-2a	A (GCA)	F (TTT)	A (GCT)	Y (TAT)	Q (CAA)	N (AAT)	T (ACA)	**M (ATG)**	S (AGT)
red fox	RF-I-32	CVP-2a/Slovakia:Presov Region/2024/red fox/RF-I-32	PX146839	CPV-2a	CPV-2a "Asian-like markers"	A (GCA)	**Y (TAT)**	A (GCT)	**I (ATT)**	Q (CAA)	N (AAT)	T (ACA)	I (ATA)	S (AGT)
red fox	RF-I-40	CVP-2a/Slovakia:Zilina Region/2024/red fox/RF-I-40	PX146840	CPV-2a	CPV-2a	A (GCA)	F (TTT)	A (GCT)	Y (TAT)	Q (CAA)	N (AAT)	T (ACA)	I (ATA)	S (AGT)
red fox	RF-RS-28	CVP-2a/Slovakia:Trnava Region/2023/red fox/RF-RS-28	PX146841	CPV-2a	CPV-2a	A (GCA)	F (TTT)	A (GCT)	Y (TAT)	Q (CAA)	N (AAT)	T (ACA)	I (ATA)	S (AGT)
red fox	RF-RS-51	CVP-2a/Slovakia:Trencin Region/2024/red fox/RF-RS-51	PX146842	CPV-2a	CPV-2a	A (GCA)	F (TTT)	A (GCT)	Y (TAT)	Q (CAA)	N (AAT)	T (ACA)	I (ATA)	S (AGT)
red fox	RF-RS-82	CVP-2a/Slovakia:Trnava Region/2025/red fox/RF-RS-82	PX146843	CPV-2a	CPV-2a	A (GCA)	F (TTT)	A (GCT)	Y (TAT)	Q (CAA)	N (AAT)	T (ACA)	I (ATA)	S (AGT)
European badger	EB-I-11	CVP-2a/Slovakia:Kosice Region/2024/European badger/EB-I-11	PX146844	CPV-2a	CPV-2a "Asian-like markers"	A (GCA)	**Y (TAT)**	A (GCT)	**I (ATT)**	Q (CAA)	N (AAT)	T (ACA)	I (ATA)	S (AGT)
European badger	EB-RS-29	CVP-2a/Slovakia:Nitra Region/2024/Europea badger/EB-RS-29	PX146845	CPV-2a	CPV-2a	A (GCA)	F (TTT)	A (GCT)	Y (TAT)	Q (CAA)	N (AAT)	T (ACA)	I (ATA)	S (AGT)
red fox	RF-I-6	CVP-2b/Slovakia:Zilina Region/2023/red fox/RF-I-6	PX146846	CPV-2b	CPV-2b	A (GCA)	F (TTT)	A (GCT)	Y (TAT)	Q (CAA)	D (GAT)	T (ACA)	I (ATA)	S (AGT)
red fox	RF-I-12	CVP-2b/Slovakia:Kosice Region/2023/red fox/RF-I-12	PX146847	CPV-2b	CPV-2b	A (GCA)	F (TTT)	A (GCT)	Y (TAT)	Q (CAA)	D (GAT)	T (ACA)	I (ATA)	S (AGT)
red fox	RF-I-15	CVP-2b/Slovakia:Zilina Region/2023/red fox/RF-I-15	PX146848	CPV-2b	CPV-2b	A (GCA)	F (TTT)	A (GCT)	Y (TAT)	Q (CAA)	D (GAT)	T (ACA)	I (ATA)	S (AGT)
red fox	RF-I-27	CVP-2b/Slovakia:Kosice Region/2023/red fox/RF-I-27	PX146849	CPV-2b	CPV-2b	A (GCA)	F (TTT)	A (GCT)	Y (TAT)	Q (CAA)	D (GAT)	T (ACA)	I (ATA)	S (AGT)

red fox	RF-I-64	CVP-2b/Slovakia:Presov Region/2025/red fox/RF-I-64	PX146850	CPV-2b	CPV-2b	A (GCA)	F (TTT)	A (GCT)	Y (TAT)	Q (CAA)	D (GAT)	T (ACA)	I (ATA)	S (AGT)
red fox	RF-I-66	CVP-2b/Slovakia:Zilina Region/2025/red fox/RF-I-66	PX146851	CPV-2b	CPV-2b	A (GCA)	F (TTT)	A (GCT)	Y (TAT)	Q (CAA)	D (GAT)	T (ACA)	I (ATA)	S (AGT)
red fox	RF-RS-2	CVP-2b/Slovakia:Trencin Region/2023/red fox/RF-RS-2	PX146852	CPV-2b	CPV-2b	A (GCA)	F (TTT)	A (GCT)	Y (TAT)	Q (CAA)	D (GAT)	T (ACA)	I (ATA)	S (AGT)
red fox	RF-RS-7	CVP-2b/Slovakia:Banska Bystrica Region/2023/red fox/RF-RS-7	PX146853	CPV-2b	CPV-2b	A (GCA)	F (TTT)	A (GCT)	Y (TAT)	Q (CAA)	D (GAT)	T (ACA)	I (ATA)	S (AGT)
red fox	RF-RS-14	CVP-2b/Slovakia:Presov Region/2023/red fox/RF-RS-14	PX146854	CPV-2b	CPV-2b	A (GCA)	F (TTT)	A (GCT)	Y (TAT)	Q (CAA)	D (GAT)	T (ACA)	I (ATA)	S (AGT)
red fox	RF-RS-39	CVP-2b/Slovakia:Presov Region/2024/red fox/RF-RS-39	PX146855	CPV-2b	CPV-2b	A (GCA)	F (TTT)	A (GCT)	Y (TAT)	Q (CAA)	D (GAT)	T (ACA)	I (ATA)	S (AGT)
red fox	RF-RS-47	CVP-2b/Slovakia:Presov Region/2024/red fox/RF-RS-47	PX146856	CPV-2b	CPV-2b	A (GCA)	F (TTT)	A (GCT)	Y (TAT)	Q (CAA)	D (GAT)	T (ACA)	I (ATA)	S (AGT)
red fox	RF-RS-64	CVP-2b/Slovakia:Kosice Region/2025/red fox/RF-RS-64	PX146857	CPV-2b	CPV-2b	A (GCA)	F (TTT)	A (GCT)	Y (TAT)	Q (CAA)	D (GAT)	T (ACA)	I (ATA)	S (AGT)
European badger	EB-I-2	CVP-2b/Slovakia:Kosice Region/2023/European badger/EB-I-2	PX146858	CPV-2b	CPV-2b	A (GCA)	F (TTT)	A (GCT)	Y (TAT)	Q (CAA)	D (GAT)	T (ACA)	I (ATA)	**I (ATT)**
European badger	EB-RS-13	CVP-2b/Slovakia:Presov Region/2023/European badger/EB-RS-13	PX146859	CPV-2b	CPV-2b	A (GCA)	F (TTT)	A (GCT)	Y (TAT)	Q (CAA)	D (GAT)	T (ACA)	I (ATA)	**I (ATT)**
European badger	EB-RS-31	CVP-2b/Slovakia:Zilina Region/2024/European badger/EB-RS-31	PX146860	CPV-2b	CPV-2b	A (GCA)	F (TTT)	A (GCT)	Y (TAT)	Q (CAA)	D (GAT)	T (ACA)	I (ATA)	**I (ATT)**
red fox	RF-F-26	CVP-2b/Slovakia:Kosice Region/2023/red fox/RF-F-26	PX146861	CPV-2b	Asian-like CPV-2b	**G (GGA)**	**Y (TAT)**	A (GCT)	**I (ATT)**	**R (CGA)**	D (GAT)	T (ACA)	I (ATA)	S (AGT)
red fox	RF-RS-5	CVP-2b/Slovakia:Kosice Region/2023/red fox/RF-RS-5	PX146862	CPV-2b	Asian-like CPV-2b	**G (GGA)**	**Y (TAT)**	A (GCT)	**I (ATT)**	**R (CGA)**	D (GAT)	T (ACA)	I (ATA)	S (AGT)
red fox	RF-RS-32	CVP-2b/Slovakia:Kosice Region/2024/red fox/RF-RS-32	PX146863	CPV-2b	Asian-like CPV-2b	**G (GGA)**	**Y (TAT)**	A (GCT)	**I (ATT)**	**R (CGA)**	D (GAT)	T (ACA)	I (ATA)	S (AGT)
red fox	RF-RS-73	CVP-2b/Slovakia:Presov Region/2024/red fox/RF-RS-73	PX146864	CPV-2b	Asian-like CPV-2b	**G (GGA)**	**Y (TAT)**	A (GCT)	**I (ATT)**	**R (CGA)**	D (GAT)	T (ACA)	I (ATA)	S (AGT)
European badger	EB-RS-8	CVP-2b/Slovakia:Kosice Region/2023/European badger/EB-RS-8	PX146865	CPV-2b	Asian-like CPV-2b	**G (GGA)**	**Y (TAT)**	A (GCT)	**I (ATT)**	**R (CGA)**	D (GAT)	T (ACA)	I (ATA)	S (AGT)
red fox	RF-RS-23	CVP-2c/Slovakia:Presov Region/2023/red fox/RF-RS-23	PX146866	CPV-2c	Asian CPV-2c	**G (GGA)**	**Y (TAT)**	A (GCT)	**I (ATT)**	**R (CGA)**	E (GAA)	T (ACA)	I (ATA)	S (AGT)

Table S5. Summary of CPE appearance in MDCK cell cultures for each CPV-positive sample.

Sample ID	Isolate	GenBank acc. number	CPV-2 variant	Sample type	Dilution	Detection of CPE
RF-F-58	CPV-2a/Slovakia:Zilina Region/2025/red fox/RF-F-58	PX146837	CPV-2a	feces	10^{-2}	3rd passage
RF-I-17	CVP-2a/Slovakia:Presov Region/2023/red fox/RF-I-17	PX146838	CPV-2a	intestine	10^{-1}	1st passage
RF-I-32	CVP-2a/Slovakia:Presov Region/2024/red fox/RF-I-32	PX146839	CPV-2a	intestine	10^{-1}	1st passage
RF-I-40	CVP-2a/Slovakia:Zilina Region/2024/red fox/RF-I-40	PX146840	CPV-2a	intestine	10^{-1}	1st passage
RF-RS-28	CVP-2a/Slovakia:Trnava Region/2023/red fox/RF-RS-28	PX146841	CPV-2a	rectal swab	10^{-1}	1st passage
RF-RS-51	CVP-2a/Slovakia:Trencin Region/2024/red fox/RF-RS-51	PX146842	CPV-2a	rectal swab	10^{-1}	2nd passage
RF-RS-82	CVP-2a/Slovakia:Trnava Region/2025/red fox/RF-RS-82	PX146843	CPV-2a	rectal swab	10^{-1}	1st passage
EB-I-11	CVP-2a/Slovakia:Kosice Region/2024/European badger/EB-I-11	PX146844	CPV-2a	intestine	10^{-1}	1st passage
EB-RS-29	CVP-2a/Slovakia:Nitra Region/2024/Europea badger/EB-RS-29	PX146845	CPV-2a	rectal swab	10^{-1}	2nd passage
RF-I-6	CVP-2b/Slovakia:Zilina Region/2023/red fox/RF-I-6	PX146846	CPV-2b	intestine	10^{-1}	1st passage
RF-I-12	CVP-2b/Slovakia:Kosice Region/2023/red fox/RF-I-12	PX146847	CPV-2b	intestine	10^{-1}	1st passage
RF-I-15	CVP-2b/Slovakia:Zilina Region/2023/red fox/RF-I-15	PX146848	CPV-2b	intestine	10^{-1}	1st passage
RF-I-27	CVP-2b/Slovakia:Kosice Region/2023/red fox/RF-I-27	PX146849	CPV-2b	intestine	10^{-1}	1st passage
RF-I-64	CVP-2b/Slovakia:Presov Region/2025/red fox/RF-I-64	PX146850	CPV-2b	intestine	10^{-1}	1st passage
RF-I-66	CVP-2b/Slovakia:Zilina Region/2025/red fox/RF-I-66	PX146851	CPV-2b	intestine	10^{-1}	1st passage
RF-RS-2	CVP-2b/Slovakia:Trencin Region/2023/red fox/RF-RS-2	PX146852	CPV-2b	rectal swab	10^{-1}	1st passage
RF-RS-7	CVP-2b/Slovakia:Banska Bystrica Region/2023/red fox/RF-RS-7	PX146853	CPV-2b	rectal swab	10^{-1}	1st passage
RF-RS-14	CVP-2b/Slovakia:Presov Region/2023/red fox/RF-RS-14	PX146854	CPV-2b	rectal swab	10^{-1}	1st passage
RF-RS-39	CVP-2b/Slovakia:Presov Region/2024/red fox/RF-RS-39	PX146855	CPV-2b	rectal swab	10^{-1}	2nd passage
RF-RS-47	CVP-2b/Slovakia:Presov Region/2024/red fox/RF-RS-47	PX146856	CPV-2b	rectal swab	10^{-1}	1st passage
RF-RS-64	CVP-2b/Slovakia:Kosice Region/2025/red fox/RF-RS-64	PX146857	CPV-2b	rectal swab	10^{-1}	2nd passage
EB-I-2	CVP-2b/Slovakia:Kosice Region/2023/European badger/EB-I-2	PX146858	CPV-2b	intestine	10^{-1}	1st passage
EB-RS-13	CVP-2b/Slovakia:Presov Region/2023/European badger/EB-RS-13	PX146859	CPV-2b	rectal swab	10^{-1}	2nd passage
EB-RS-31	CVP-2b/Slovakia:Zilina Region/2024/European badger/EB-RS-31	PX146860	CPV-2b	rectal swab	10^{-2}	1st passage

RF-F-26	CVP-2b/Slovakia:Kosice Region/2023/red fox/RF-F-26	PX146861	CPV-2b	feces	10^{-2}	2nd passage
RF-RS-5	CVP-2b/Slovakia:Kosice Region/2023/red fox/RF-RS-5	PX146862	CPV-2b	rectal swab	10^{-1}	2nd passage
RF-RS-32	CVP-2b/Slovakia:Kosice Region/2024/red fox/RF-RS-32	PX146863	CPV-2b	rectal swab	10^{-1}	1st passage
RF-RS-73	CVP-2b/Slovakia:Presov Region/2024/red fox/RF-RS-73	PX146864	CPV-2b	rectal swab	10^{-1}	2nd passage
EB-RS-8	CVP-2b/Slovakia:Kosice Region/2023/European badger/EB-RS-8	PX146865	CPV-2b	rectal swab	10^{-1}	1st passage
RF-RS-23	CVP-2c/Slovakia:Presov Region/2023/red fox/RF-RS-23	PX146866	CPV-2c	rectal swab	10^{-1}	2nd passage

RF – red fox; EB – European badger; F – fecal sample; I - small intestine; RS – rectal swab.
